# IL-10-Secreting CD8^+^ T Cells Specific for Human Cytomegalovirus (HCMV): Generation, Maintenance and Phenotype

**DOI:** 10.3390/pathogens11121530

**Published:** 2022-12-13

**Authors:** Sarah E. Jackson, George X. Sedikides, Veronika Romashova, Georgina Okecha, Ester B. M. Remmerswaal, Frederike J. Bemelman, John H. Sinclair, Mark R. Wills

**Affiliations:** 1 Department of Medicine, Cambridge Institute of Therapeutic Immunology and Infectious Disease, Cambridge Biomedical Campus, School of Clinical Medicine, University of Cambridge, Cambridge CB2 2QQ, UK; 2 Department of Experimental Immunology, Amsterdam Infection and Immunity Institute, Amsterdam UMC, University of Amsterdam, 1019 Amsterdam, The Netherlands; 3 Academic Medical Centre, Renal Transplant Unit, Division of Internal Medicine, Amsterdam UMC, University of Amsterdam, 1019 Amsterdam, The Netherlands

**Keywords:** human cytomegalovirus (HCMV), CD8^+^ T cells, IL-10 secretion, immunomodulation, T regulatory cells

## Abstract

HCMV-specific CD8^+^ T-cells are potent anti-viral effector cells in HCMV infected individuals, but evidence from other viral infections suggests that CD8^+^ T-cells can also produce the immunomodulatory cytokine IL-10. In this work we show that there are HCMV-specific IL-10 CD8^+^ T-cell responses in a cohort of individuals aged 23–76 years of age, predominantly directed against the HCMV proteins known to be expressed during latent infections as well as towards the proteins US3 and pp71. The analysis of HCMV-specific responses established during primary infection has shown that the IL-10 responses to US3 and pp71 HCMV proteins are detectable in the first weeks post infection, but not the responses to latency-associated proteins, and this IL-10 response is produced by both CD8^+^ and CD4^+^ T-cells. Phenotyping studies of HCMV-specific IL-10^+^ CD8^+^ T-cells show that these are CD45RA^+^ effector memory cells and co-express CD28 and CD57, however, the expression of the inhibitory receptor PD-1 varied from 90% to 30% between donors. In this study we have described for the first time the HCMV-specific IL-10 CD8^+^ T-cell responses and have demonstrated their broad specificity and the potential immune modulatory role of the immune response to HCMV latent carriage and periodic reactivation.

## 1. Introduction

Human cytomegalovirus (HCMV) is a common infection worldwide which does not cause obvious disease in healthy individuals and induces a robust immune response incorporating secretory and cellular responses to control the lytic virus infection [1,2]. Despite this immune response, the virus is not cleared but is able to establish a latent infection which persists for the lifetime of the host in bone marrow resident CD34^+^ cells and their myeloid derivatives. Latent HCMV infection is defined as the absence of infectious virion production, which contrasts with lytic infection where there is broad transcription of all viral genes. Several viral genes have been identified as being expressed during latent infection in the absence of immediate early (IE) gene expression, including UL138, LUNA (UL81-82as), US28, UL111A (vIL-10) and UL144 [3], and recent advances in the analysis of viral gene transcription have identified many other viral genes which may be expressed at low levels during a latent infection in myeloid cells and their progenitors [4,5]. The detection of genes usually expressed during a lytic infection in the cell types associated with a repressed latent infection is likely to be a result of a new infection in these cells, which decreases over time as the latent infection is established [3]. The latent virus infection, however, does not remain quiescent and periodically reactivates upon differentiation of myeloid cells [3,6] leading to antigenic stimulation and the expansion of HCMV-specific memory immune responses and resulting in distinct T cell memory and effector populations [7,8]. It is well established that CD8^+^ T cells play a key role in controlling initial infection and subsequent reactivation events [9]; indeed, HCMV-specific CD8^+^ T cell responses to the proteins pp65 and IE1 often dominate the CD8^+^ T cell repertoire [10,11]. The HCMV-specific CD8^+^ T cell response has been extensively characterized and there are distinct differentiated memory CD8^+^ T cell populations observed, consisting of re-expression of the CD45RA isoform, upregulation of the natural killer associated receptors, such as KLRG1, CD57, NKG2A and CD56, and loss of the costimulatory receptors CD27 and CD28 [12]. The IFNγ-secreted responses to a range of HCMV proteins have been demonstrated in numerous studies [11,13,14]. The CD8^+^ T cell response to HCMV has been shown to be polyfunctional with multiple inflammatory cytokines being secreted and strong cytotoxic functions [15,16,17], as well as in vitro direct anti-viral capacity [13,18]. The polyfunctional nature of the CD8^+^ T cell response has been shown to be important in controlling HCMV reactivation, as a decrease in polyfunctional responses results in increased reactivation in sepsis patients [19].

There is a well-established manipulation of the immune response by HCMV during lytic infection resulting from the expression of a number of virally encoded immune evasion molecules. These help the evasion of antigen presentation, dysregulate host interferon response pathways, prevent natural killer cell receptor recognition and act as receptor sinks for inflammatory cytokines [20]. However, it is apparent that there is also manipulation of the host immune response during latent infection via the expression of viral microRNAs and viral proteins which modify the latently infected cell and manipulate the cellular microenvironment [21]. The viral proteins expressed during latency that can control the immune response include US28, a G-protein coupled receptor which downregulates interferon-inducible genes [22], and the latency-associated splice product of the UL111A ORF, a viral homologue of IL-10 (vIL-10), which can manipulate CD4^+^ T cell recognition via the downregulation of MHC Class II molecules on myeloid cells [23]. As well as producing a viral homologue of IL-10, HCMV-latent carriage in both CD34^+^ cells and monocytes also alters the cellular secretome by increasing the production of cellular IL-10 (cIL-10), which inhibits anti-viral CD4^+^ effector functions [24,25]. This modulation of the CD4^+^ T cell function also extends to HCMV-specific CD4^+^ T cells which have a regulatory cell phenotype or secrete cIL-10 [14,26,27,28,29,30,31], and have been observed to play an important role in resolving the complications from HCMV infection in hematopoietic stem cell transplant patients [32]. Whilst CD4^+^ regulatory T cells have been extensively studied, the analysis of CD8^+^ regulatory T cells is a neglected area of research [33] and is considered to be controversial in part due to the limited number of studies published showing the immunoregulatory functions of CD8^+^ T cells [34].

CD8^+^ regulatory T cells are heterogenous in nature and have been described in autoimmune, allergy and transplantation conditions. Phenotypically, three major groups have been described in humans [35], these are (i) CD28negative CD39^+^ CD8^+^ T cells which have been described in HIV infection [36], (ii) CXCR3^+^ CD45RA^−^ CD62L^+^ CD8^+^ T cells (CD122^+^ in rodent models) that suppress IFNγ production by other T cells via IL-10 production [37] and (iii) PD-1^+^ CD8^+^ T cells that secrete IL-10 which have been described in HIV infection [38], hepatitis B (HBV) infection [39] and chronic hepatitis C (HCV) infection [40]. There are limited reports on IL-10 secretion by CD8^+^ T cells in HCMV infection but liver transplant recipients who progressed to CMV disease had increased expression of PD-1 on both CD4^+^ and CD8^+^ T cells and increased IL-10 in their plasma [41]. Similarly, HCMV-specific CD8^+^ T cells in HBV-infected patients show increased PD-1 expression and loss of IFNγ production [42].

Previously, we demonstrated that there are CD4^+^ T cell IL-10 responses to the latency-associated proteins UL138 and LUNA [31] and the HCMV proteins US3 and pp71 [43]. However, the frequency and magnitude of these IL-10 responses are not affected by the length of viral carriage using donor age as a surrogate for this [14]. Earlier studies did not detect many CD8^+^ T cell responses to the latency-associated proteins UL138 and US28 [11] and only below-threshold low frequency CD8^+^ T cell responses were observed by ELISpot to UL138 and LUNA peptide pools [31]. In our survey of the T cell responses to 11 HCMV proteins, including the latency-associated proteins UL144, US28, vIL-10, LUNA and UL138, we measured CD8^+^ T cell IFNγ responses in many donors in response to UL144 and US28, as well as a response in 30% of donors to LUNA and UL138 [14].

Using the same cohort of donors, we have now analyzed the CD4-depleted peripheral blood mononuclear cells (PBMC) (enriched for CD8^+^ T cells) for CD8^+^ IL-10 responses to HCMV protein peptide pool stimulation. Due to an increased nonspecific background production of IL-10 by the CD8^+^ T cell-enriched PBMC population compared to the CD4^+^ T cell-enriched populations, 49 donors from the cohort were analyzed. The results show that the majority of the donor cohort made an IL-10 response to one or more HCMV protein peptide pools, with IL-10 responses to pp71, US28 and US3 predominating. The IL-10 secretion by CD3^+^ CD8^+^ T cells in response to HCMV protein stimulation was confirmed by a separate analysis using intracellular cytokine staining for IL-10 and IFNγ. Longitudinal samples from seven primary HCMV infections (donor seropositive, recipient seronegative (D^+^R^−^) kidney transplant patients) were also studied for the IL-10 responses to a broad range of pooled HCMV proteins. The IL-10 responses generated by both CD8^+^ and CD4^+^ T cells were present within 1 month of infection in response to a mix of pp71 and US3 proteins, but not in response to latency associated proteins, despite IFNγ-specific responses being detected from early timepoints post infection.

The identification of the CD8^+^ T cell subset producing IL-10 in response to HCMV protein stimulation was undertaken by flow cytometry phenotyping of IL-10-secreting HCMV-specific CD8^+^ T cells for memory, differentiation and regulatory associated markers. We observed HCMV-specific IL-10-secreting CD8^+^ T cells which varied between donors, with CMV319 having the majority of the IL-10-positive population expressing PD-1 and CD39 whereas donor CMV332 only had 30% of the IL-10-secreting population expressing PD-1. It is clear, however, that the frequency of IL-10-secreting T cells in peripheral blood fluctuates over time and, of note, we observe that there are CD8*+* T cell IL-10 responses to a diverse set of HCMV proteins which may be present in tissue sites, such as the bone marrow, which results in these immunomodulatory cells generating microenvironments which could influence the persistence of HCMV latency and reactivation.

## 2. Materials and Methods

### 2.1. Donor Sample and Ethics Statement

Healthy CMV-seropositive and negative donors were recruited locally with ethical approval from the Addenbrookes National Health Service Hospital Trust institutional review board (Cambridge Research Ethics Committee); informed written consent was obtained from all volunteers in accordance with the Declaration of Helsinki (Cambridgeshire 2 REC 97/092). Twenty-two HCMV-seropositive donors (5/17 female/male) aged 23–77 years were recruited. A second healthy donor cohort was recruited from the National Institute of Health Research (NIHR) Cambridge BioResource. Ethical approval was obtained from University of Cambridge Human Biology Research Ethics Committee. Informed written consent was obtained from all donors in accordance with the Declaration of Helsinki (HBREC.2014.07). A cohort of 46 HCMV-seropositive donors aged 24–76 years and 3 seronegative donors aged 37–71 years were included in this ARIA study. The CMV serostatus of all donors was confirmed by a serological assessment of CMV IgG levels using the Captia CMV IgG EIA test (Trinity Biotech, Bray, Ireland) following the manufacturer’s instructions. Details of the ARIA donor cohort studied are provided in Table S1.

Seven seropositive donors to seronegative recipient (D^+^R^−^) kidney transplant patients were recruited by the Academisch Medisch Centrum (AMC), Amsterdam, The Netherlands, who experienced a primary HCMV infection post-transplantation. The transplants took place between 2003 and 2009. Ethical permission was granted by the Medical Ethics Committee of the AMC, Amsterdam and all patients gave informed written consent in accordance with the Declaration of Helsinki. The donor and recipient serostatus were defined using a microparticle enzyme immunoassay as previously described [44]. The PBMC were collected at multiple time points after transplantation, with subsequent samples collected at varying time points up to a maximum of 158 weeks post-transplantation, isolated by density centrifugation and cryopreserved [44]. Virus load monitoring was performed by quantitative PCR (qPCR) as previously described [45]. The PBMC were a kind gift from Professors I.J.M. ten Berge and R.A.W. van Lier (Amsterdam Renal Transplant Unit). Patient immunosuppression, viral treatment and seroconversion data are summarized in Table S2.

Paired bone marrow mononuclear cells and peripheral blood mononuclear cells from a HCMV-seropositive donor were purchased from AllCells (distributed by Lonza, Slough, UK).

### 2.2. Peripheral Blood Mononuclear Cell Isolation

Peripheral blood mononuclear cells were isolated from heparinized blood samples using Lymphoprep (Axis-shield, Oslo, Norway) or Histopaque-1077 (Sigma-Aldrich, Merck Life Science UK Ltd, Dorset, UK) density gradient centrifugation.

### 2.3. HCMV ORF Peptide Mixes

Seven HCMV ORF-encoded proteins (UL82 (pp71), UL122 (IE2), UL123 (IE1), US3, UL138, US28 and UL111A (vIL-10)) were selected and peptide libraries comprising consecutive 15mer peptides overlapping by 10 amino acids were synthesized by ProImmune PEPScreen (Oxford, UK) from sequences detailed in the Sylwester et al. study [11]. A further 3 HCMV ORF-encoded proteins’ (UL83 (pp65) [46], UL144 (incorporating known strain variants) [47] and LUNA (UL81-82as) [31]) 15mer peptide libraries were synthesized by JPT Peptide Technologies GmbH (Berlin, Germany). The individual lyophilized peptides from each ORF library were reconstituted and used as previously described [13].

### 2.4. ARIA Study Dual FluoroSpot Assays

The peripheral blood mononuclear cells were depleted of CD4^+^ T cells by MACS using anti-CD4^+^ direct beads (Miltenyi Biotec, Bisley, UK), according to the manufacturer’s instructions and separated using an AutoMACS Pro (Miltenyi Biotec). The efficiency of depletion was determined by staining cells with a CD3-FITC, CD4-PE and CD8-PerCP-Cy5.5 antibody mix (BioLegend, San Diego, CA, USA; full details in Table S3) and analyzed by flow cytometry. The depletion in this manner resulted in a mean of 8.6% residual CD4^+^ T cells [14]. A total of 2 × 10^5^ PBMC depleted of CD4^+^ T cells suspended in X-VIVO 15 (Lonza, Slough, UK) supplemented with 5% human AB serum (Sigma-Aldrich) were incubated in pre-coated FluoroSpot plates (human IFNγ and IL-10 FluoroSpot (Mabtech AB, Nacka Strand, Sweden)) in triplicate with 10 ORF-mix peptides (final peptide concentration 2 μg/mL/peptide) and an unstimulated and positive control mix (containing anti-CD3 (Mabtech AB), Staphylococcus enterotoxin B, phytohemagglutinin, pokeweed mitogen and lipopolysaccharide (all Sigma-Aldrich)) at 37 °C in a humidified CO_2_ atmosphere for 48 h. The cells and medium were decanted from the plate and the assay was developed following the manufacturer’s instructions. The developed plates were read using an AID iSpot reader (Oxford Biosystems, Oxford, UK) and counted using AID EliSpot v7 software (Autoimmun Diagnostika GmbH, Straberg, Germany) using distinct counting protocols for IFNγ and IL-10 secretion. The donor results were quality controlled as previously described [14], and the presented data is corrected for background cytokine secretion. A comparison between the distribution of the response from the HCMV-seropositive and seronegative donors to all the HCMV proteins and the positive control was utilized to determine the threshold of the positive response of 100 sfu/million for both the IFNγ and IL-10 responses.

### 2.5. Intracellular Cytokine Staining of CD8^+^ T cells

The polyfunctional CD8^+^ T cell responses to HCMV ORF peptides were determined using intracellular cytokine staining. In total, 2–3 × 10^6^ PBMC were resuspended in 200 μL X-VIVO 15 in polypropylene FACS tubes with 250 μL of either X-VIVO 15 only, positive control mixture or HCMV ORF mixes added. The cells were incubated overnight at 37 °C after 20 h monensin (BioLegend) was added at a 1:100 dilution and incubated for a further 4 h. The cells were washed in FACS wash buffer (PBS without calcium and magnesium supplemented with 0.5% bovine serum albumin, 2mM EDTA (Thermo Fisher Scientific, UK) and 2 mM sodium azide) and stained with antibodies (detailed Table S3) that detect surface antigens (CD14 BV510, CD19 BV510 and CD3 BV650 (BioLegend)) and a live cell discriminator (LIVE/DEAD Fixable Aqua Dead Stain Kit (Thermo Fisher Scientific)) for 20 min at 4 °C. The cells were then fixed using 100 μL of solution A (FIX & PERM kit (Nordic-MUbio)) for a further 20 min at 4 °C. The cells were washed and resuspended in a mix of intracellular antibodies (CD69 Pacific Blue, 4-1BB PE-Cy5, IL-10 PE, CD4 BV605, CD8 BV570 (Biolegend) and IFNγ BV786 (BD Biosciences, Wokingham, UK)) in solution B (FIX & PERM) for 30 min at 4 °C. The cells were washed in FACS wash buffer and resuspended in a final concentration of 2% paraformaldehyde (PFA); the samples were acquired on a BD LSR-Fortessa (BD Biosciences) using FACS Diva software and analyzed with FlowJo software (Treestar, BD Biosciences); the samples were compensated and transformed using the software and then analyzed using the gating strategy shown in Figure S1. A HCMV positive CD8^+^ T cell response was determined by subtracting the 4-1BB^+^/CD69^+^ unstimulated response (background) from the HCMV protein stimulated 4-1BB^+^/CD69^+^ response, additionally donor samples with low positive control responses (background corrected values less than 1%) were excluded from the analysis. The positive response threshold of 0.25% 4-1BB^+^/CD69^+^ CD8^+^ T cells was calculated as the median plus two-fold standard error of the mean (SEM) of all the negative controls.

### 2.6. FluoroSpot Analysis of Longitudinal Kidney Transplant Samples

The frozen longitudinal samples were removed from liquid nitrogen storage and the rapidly warmed cells were immediately diluted in excess defrosting media (warmed DMEM (Sigma-Aldrich) supplemented with 10 U/mL benzonase (Merck Millipore)). The cells were washed by centrifugation for 10 min at 300× *g* before being resuspended in X-VIVO 15 supplemented with 10 U/mL benzonase and incubated for 1 h at 37 °C. The cells were again washed by centrifugation for 10 min at 300× *g* and resuspended in X-VIVO-15 and rested overnight at 37 °C prior to use. Pre-coated human IFNγ, IL-10 and TNFα FluoroSpot plates or non-coated IFNγ and IL-10 FluoroSpot-FLEX kits (Mabtech AB) were used with a minimum of 50,000 cells per well in triplicate of the total PBMC or PBMC depleted of either CD4^+^ or CD8^+^ T cell using anti-CD4 or anti-CD8 direct beads as previously described. The patient samples were stimulated with HCMV ORF peptide pools grouped together as latency associated proteins (LAT: UL138, US28, LUNA, vIL-10), pp65 and UL144, IE1 and IE2, pp71 and US3 and gB protein with an unstimulated and positive control mix (as before) for 37 °C for 48 h. The cells and media were decanted and the assay was developed following the manufacturer’s protocol and read and analyzed as described above. The input CD3^+^ T cells were enumerated by staining the samples with a mix of CD3-FITC, CD4-PE and CD8-PerCPCy5.5 (BioLegend) and the LIVE/DEAD Fixable Far Red Dead Stain Kit (Thermo Fisher Scientific) and a known volume run on a BD Accuri C6^+^ (BD BioSciences) and analyzed with FlowJo software. The results are summarized in Figure S4.

### 2.7. Phenotyping of IL-10-Secreting CD8^+^ T Cells

The production of IL-10 by CD8^+^ T cells in response to HCMV peptide stimulation was performed using the IL-10 Secretion Assay – Detection Kit (PE) (Miltenyi Biotec) following the manufacturer’s protocol. Briefly, PBMC in TexMACS media were stimulated in a 96-well flat bottom plate at a concentration of 1.5 × 10^6^ cells per well with 2 wells per stimulation condition of unstimulated, positive control mix and 4 HCMV peptide pools (pp65 and UL144; IE1 and IE2; latency associated proteins (LAT); pp71 and US3) for 6 h at 37 °C. The cells were collected and washed in cold FACS wash buffer by centrifugation at 300× *g* for 10 min at 4 °C, and then resuspended in a cold medium. An IL-10 catch reagent was added, mixed and the cells were incubated for 5 min on ice. Following the addition of excess warm media, the cells were incubated for 45 min at 37 °C using constant rotation provided by a MACSmixer (Miltenyi Biotec). The cells were then washed with cold buffer and stained with the IL-10 detection antibody (PE) with the LIVE/DEAD Fixable Aqua dead stain, CD14-BV510, CD19-BV510, CD3-BV650, CD8-BV570, CD4-BV605, PD-1-BV421, CD57-PE-Dazzle 594, CD137 (4-1BB)-PE-Cy5, CD40L-PerCP-Cy5.5, CD45RA-PE-Cy7, CD27-APC, CD28-Alexa Fluor 700 (Biolegend) and CD39-BUV737 (BD Biosciences) (full details Table S3) for 30 min at 4 °C. Following a wash in cold FACS wash buffer, the cells were fixed using Fluoro-fix (BioLegend) and then samples were acquired on an LSR Fortessa with FACS Diva software. Single-color compensation controls using ArC™ and AbC™ compensation beads (Thermo Fisher) were also prepared and run with the samples. The acquired data was cleaned up and processed using FlowJo v10.8.1 as outlined in Supplementary Figure S5. Following the compensation and transformation of the acquired samples the pre-processed samples were analyzed (data is shown in Figure S6). The unstimulated and latency-associated transcript samples for each donor were downsampled and the CD8^+^-gated samples were concatenated to enable dimensionality reduction t-distributed stochastic neighbor embedding (tSNE) visualization to be performed using the embedded tSNE function [48,49], and a clustering algorithm FlowSOM (using a plugin within FlowJo v10.8.1) was used to transform the data [50].

### 2.8. FluoroSpot Analysis of PD-1-Depleted CD8^+^ T Cells

The CD14 monocytes were isolated from the PBMC using anti-CD14 direct beads (Miltenyi Biotec). The CD8^+^ T cells were isolated from the CD14-negative fraction with a CD8^+^ T cell isolation kit (Miltenyi Biotec). The PD-1^+^ CD8^+^ T cells were depleted from the purified CD8^+^ T cells using anti-PD-1 FITC (Clone EH12.2H7; BioLegend) and anti-FITC Microbeads (Miltenyi); all the magnetic separations were run on an AutoMACS Pro using the appropriate programs for each separation. The non-coated IFNγ and IL-10 FluoroSpot-FLEX kits (Mabtech) were run with 100,000 isolated CD8^+^ T cells (PD-1 non-depleted and depleted) and 50,000 CD14^+^ monocytes as antigen-presenting cells in triplicate for each stimulation in the TexMACS medium (Miltenyi Biotec). The unstimulated and positive control samples were run with the HCMV-peptide pool mixes (pp65 and UL144; IE1 and IE2; latency associated proteins (LAT); pp71 and US3) for 48 h at 37 °C. FluoroSpot plates were developed and analyzed as described. The purity of the CD8^+^ T cell isolation and PD-1 depletion were determined using CD4-FITC or PD-1 FITC, CD8-PE, CD3-PerCPCy5.5 (BioLegend—Table S3) and the LIVE/DEAD Fixable Far Red Dead Stain kit and run on a BD Accuri C6^+^ and analyzed with FlowJo software; the results are shown in Figure S8.

### 2.9. FluoroSpot Analysis of Paired Bone Marrow and Peripheral Blood Mononuclear Cells

The frozen bone marrow and peripheral blood mononuclear cells (BMMNC and PBMNC) were removed from liquid nitrogen storage and defrosted as previously described prior to an overnight rest before use. Pre-coated human IFNγ, IL-10 and TNFα FluoroSpot plates (Mabtech AB) were used with a minimum of 30,000 cells per well in triplicate of BMMNC and 150,000 cells per well in triplicate of PBMNC depleted of CD4^+^ or CD8^+^ T cells using anti-CD4 or anti-CD8 direct beads as previously described. The samples were stimulated with 10 HCMV ORF peptide pools (UL138, US28, vIL-10, LUNA, UL144, pp65, IE1, IE2, pp71 and US3) with an unstimulated and positive control mix (as before) for 37 °C for 48 h. The assay was developed following the manufacturer’s protocol and read and analyzed as described above. The input CD3^+^ T cells were enumerated by staining the samples with a mix of CD3-FITC, CD4-PE and CD8-PerCPCy5.5 (BioLegend) and the LIVE/DEAD Fixable Far Red Dead Stain kit (Thermo Fisher Scientific) and a known volume run on a BD Accuri C6^+^ (BD BioSciences) and analyzed with FlowJo software. Typically, CD3^+^ T cells represent about 30% of BMMNC and 60% of PBMNC lymphocytes; in this experiment BMMNC CD8^+^ T cells were enriched to 62.2% of CD3^+^ T cells and PBMNC to 66.1% from 25% and 28%, respectively.

### 2.10. Statistics

The statistical analysis and graphical presentation were performed using GraphPad Prism version 9 for Windows (GraphPad Software, San Diego, CA, USA). The correlation between age and T cell responses to CMV stimulation was assessed by the Spearman rank correlation for non-normally distributed data.

## 3. Results

### 3.1. HCMV-Specific CD8^+^ T Cells Can Produce IL-10

**Figure 1:**
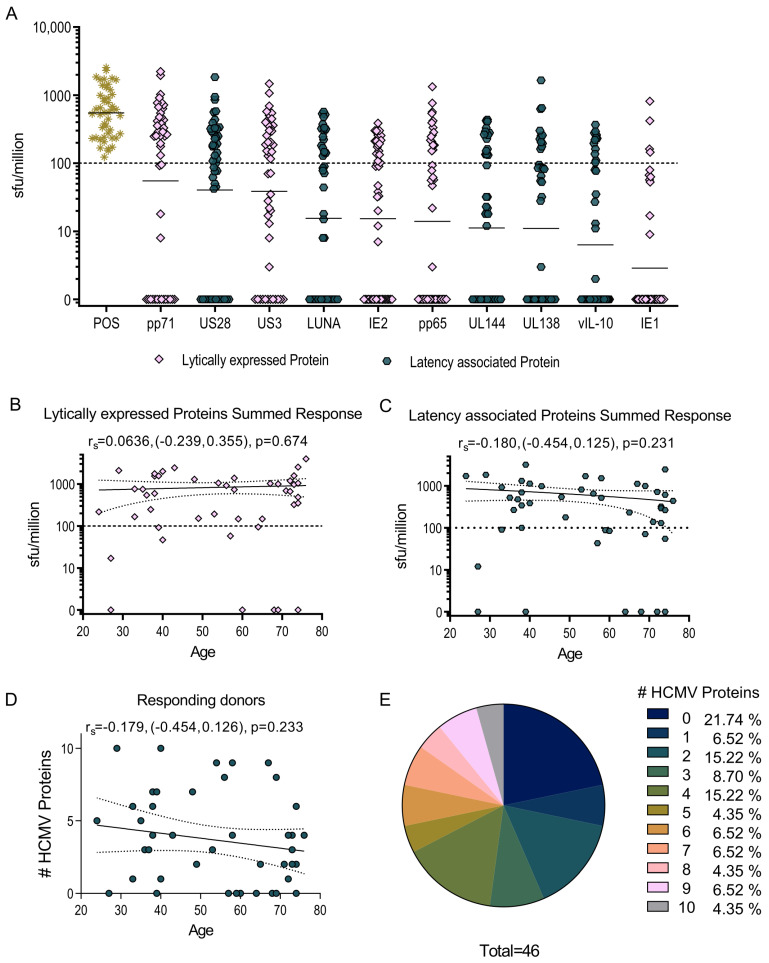
HCMV-specific CD8^+^ T cells produce IL-10 in response to HCMV protein stimulation. The IL-10-secreting CD8^+^ T cell response to 10 HCMV proteins UL138, US28, LUNA, UL144, vIL-10 (latency-associated proteins, dark blue colored points), pp71, US3, pp65, IE2, IE1 (lytically expressed proteins, peach colored points) and the positive control was measured in a cohort of 46 HCMV-seropositive donors using a IFNγ and IL-10 dual FluoroSpot technique. The results were converted to sfu/million T cells with background counts subtracted and the positive response threshold of 100 sfu/million is indicated (dashed line) on the graph (**A**) with the geo-mean of each protein response shown as a line. The IL-10 response to the lytically expressed HCMV proteins (**B**) and the latency-associated proteins (**C**) is shown as a correlation of donor age with the cumulative size of the donor response. The breadth of the IL-10 CD8^+^ response (the number of HCMV proteins to which each donor had an IL-10 response above-threshold) was enumerated and plotted by age (**D**). The correlation of the response with age was analyzed using the Spearman rank correlation (Spearman r_s_ with 95% confidence intervals (CI) and p value are indicated with the line of best fit (solid) and 95% CI (dotted lines) also shown on the graphs). The proportion of donors responding to one or more of the HCMV proteins are summarized as a pie chart with the key to segments shown (**E**).

In a previous study we demonstrated that CD4^+^ T cells specific for a number of HCMV protein peptide pools could produce IL-10, and that these T cells were biased towards pp71 and US3 as well as the latency-associated proteins US28, LUNA, vIL-10 and UL138 [14]. We have now analyzed whether there are HCMV-specific IL-10-producing CD8^+^ T cells within this study cohort. CD4^+^ T cell depleted PBMC from 49 HCMV seropositive donors (Table S1) was stimulated with 10 HCMV protein peptide pools spanning the ORFs UL144, pp71, US3, pp65, IE1, IE2, UL138, US28, LUNA and vIL-10 in a dual IFNγ and IL-10 FluoroSpot assay to quantify the responses. The results clearly demonstrate that HCMV-specific CD8^+^ T cells can produce IL-10 (Figure 1A) and there was no significant correlation between the IL-10 responses to the lytically expressed and latency-associated groups for protein and donor age (Figure 1B,C). The proportion of donors producing an IL-10 response to each HCMV protein peptide pool are summarized in Table S4 with the responder frequency of above-threshold responses being highest for pp71 (60.9%), US28 (54.3%) and US3 (52.2%) and lowest for IE1 (8.7%) stimulation. Overall, 78.2% of the donors generated a positive IL-10 response to one or more HCMV protein and two donors produced an IL-10 response to all 10 HCMV ORFs. The ability to generate a broad IL-10 response to HCMV proteins was also not affected by donor age (Figure 1D,E).

**Figure 2:**
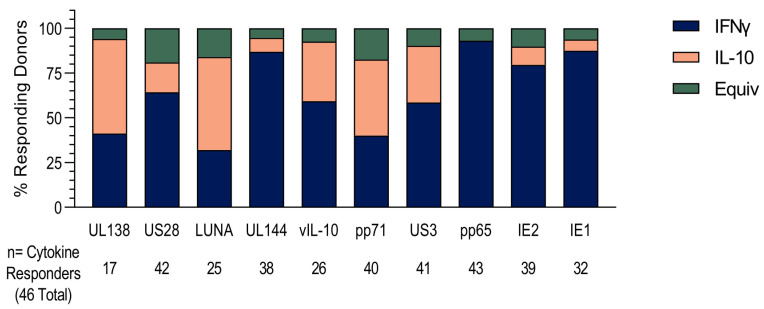
Dominant cytokine response to HCMV protein stimulation. The dominant cytokine response to HCMV protein stimulation of CD8^+^ T cells was calculated. A dominant response was defined as over 55% of the total CD8^+^ T cell-positive response; the number of donors generating a dominant IFNγ response (navy bars) or IL-10 response (peach bars) or an equivocal response (green bars) are shown as stacked bars for the 10 HCMV proteins. Also indicated are the total number of responding donors (either cytokine) to each of the 10 HCMV proteins.

Next, we investigated the cytokine composition of each donor’s CD8^+^ T cell response to HCMV protein stimulation, and whether it comprised the production of IFNγ or IL-10 or both by individual T cells. The majority cytokine response of each donor, whether IFNγ or IL-10, was tallied and is presented in Figure 2. The data shows that the responses to UL138, LUNA and pp71 proteins were dominated by IL-10 production over IFNγ and that almost 30% of the donors that responded to US3 and vIL-10 were IL-10-biased. Together with the magnitude and proportion data (Figure 1), this analysis shows that the CD8^+^ T cell response to the latency-associated proteins UL138, US28, LUNA and vIL-10 and the lytically expressed proteins pp71 and US3 in HCMV-positive donors is often composed of IL-10-secreting T cells and that these are predominantly a separate subset from IFNγ-producing cells specific for the same peptide pool.

**Figure 3:**
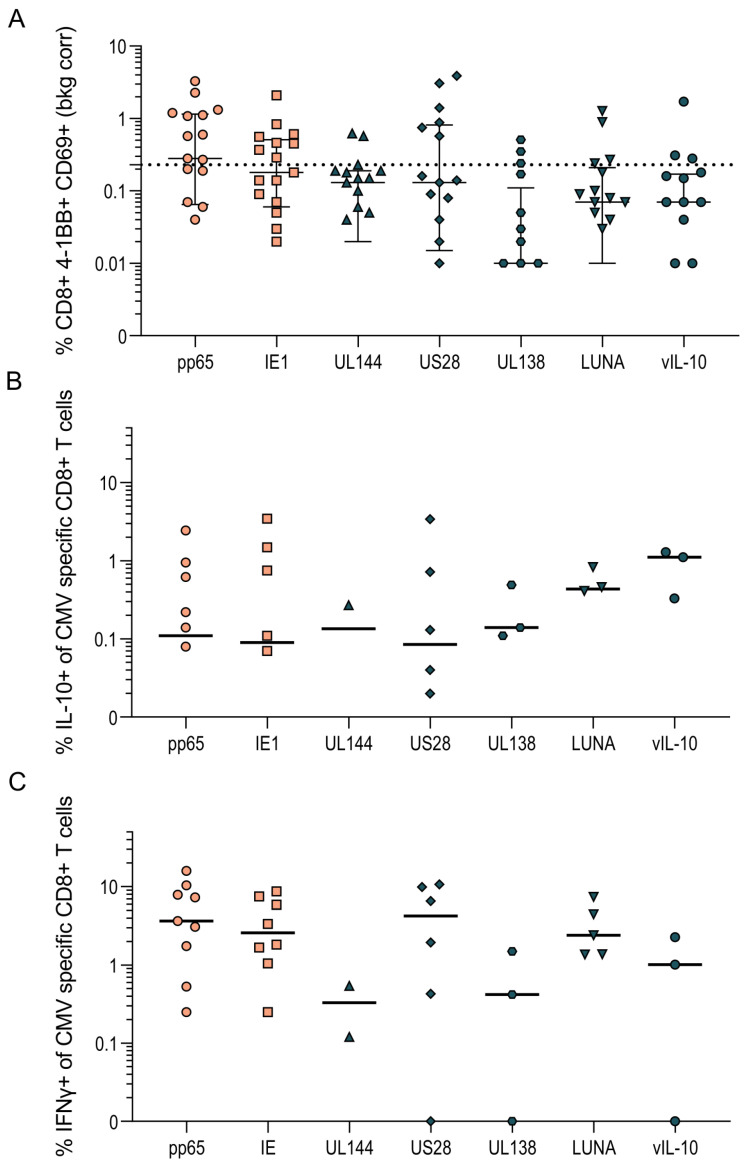
PBMC were stimulated overnight with HCMV peptide pools in the presence of monensin. The identification of HCMV-specific CD8^+^ T cell-positive responses was determined by the expression of 4-1BB and/or CD69 upregulation above background and the percentage of IL-10 and IFNγ response in the positive cells measured. The background-corrected percentages of 4-1BB^+^ and/or CD69^+^ CD8^+^ T cells to each HCMV peptide pool are shown with the positive threshold of 0.25% (dotted line) indicated (**A**). The percentage of IL-10 (**B**) and IFNγ (**C**) secretion of HCMV-specific CD8^+^ T cells from 17 donors to the seven HCMV proteins pp65, IE (peach filled shapes, lytically expressed proteins), UL144, US28, UL138, LUNA and vIL-10 (dark blue filled shapes, latency-associated proteins) are shown.

These assays were performed on PBMC depleted of CD4^+^ T cells which, though enriched for CD8^+^ T cells, also contain NK cells, B cells and γδ T cells. To confirm that the IL-10 responses we had identified were from the CD8^+^ T cell population, intracellular cytokine staining of 17 HCMV-seropositive donors was performed, with the results summarized in Figure 3. The CD8^+^ T cell population was identified by gating live, single lymphocytes and then CD3^+^ T cells and the CD8^+^ subset of CD3^+^ T cells (outlined in Figure S1). HCMV-specific CD8^+^ T cell responses were defined by the upregulation of the activation markers 4-1BB and CD69 above background; a positive response was deemed greater than 0.25% once the background was corrected (Figure 3A). The proportion of activated CD8^+^ T cells producing IFNγ and IL-10 in response to stimulation with HCMV protein pools were then enumerated. In donors with an above-background HCMV-specific response to the proteins pp65 and IE (lytically expressed proteins) and UL144, US28, UL138, LUNA and vIL-10 (latency-associated proteins), the percentage of IL-10-producing cells was collated and is presented in Figure 3B, with the percentage of IFNγ-producing cells shown in Figure 3C. These results clearly show that CD3^+^ CD8^+^ T cells produce IL-10 in response to stimulation with HCMV protein peptides.

### 3.2. US3 and pp71 IL-10 CD8^+^ T cell Responses Arise Early in Primary Infection

**Figure 4:**
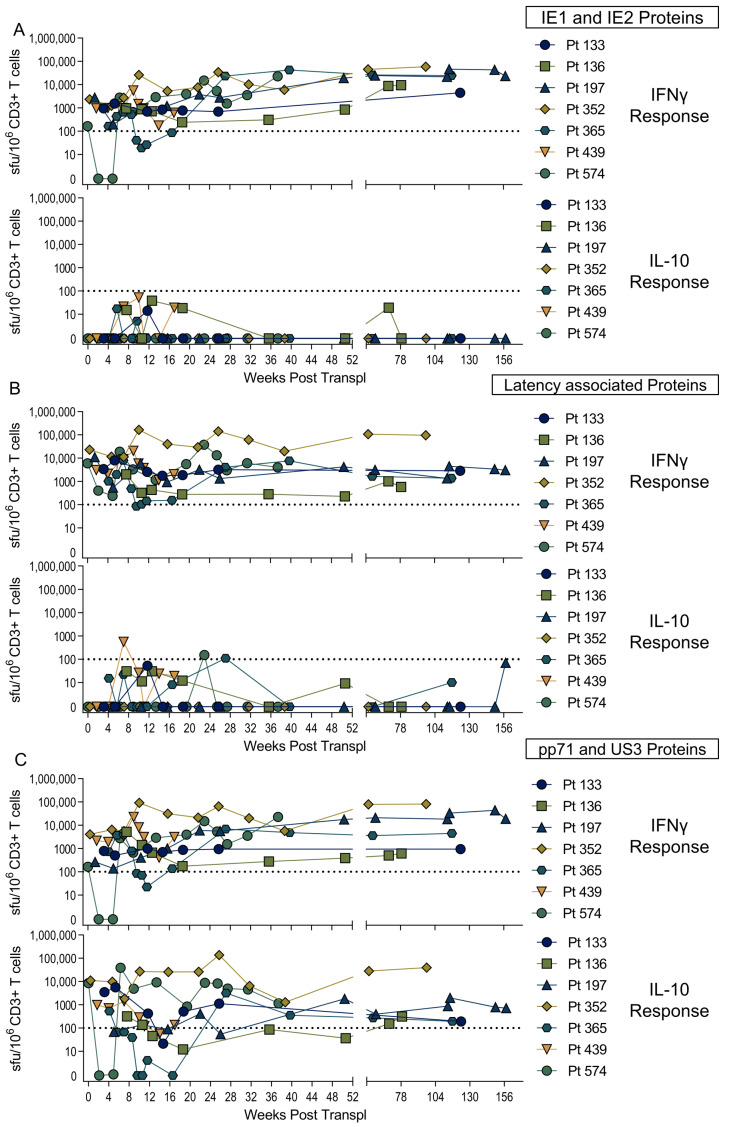
HCMV-specific IL-10 responses arise early in primary infection. A cohort of seven D^+^R^−^ transplant patients who had primary HCMV infection post transplantation was recruited by the Academisch Medisch Centrum (AMC) Amsterdam. PBMC were collected at multiple time points after transplantation and analyzed for T cell secretion of IFNγ and IL-10 by FluoroSpot in response to pooled HCMV protein stimulations. The background corrected responses of the total PBMC from the seven patients are illustrated showing the IFNγ and IL-10 T cell responses over the weeks post-transplant for IE1 and IE2 stimulation (**A**), UL138, US28, LUNA and vIL-10 (latency associated proteins) stimulation and (**B**) pp71 and US3 stimulation, with (**C**) the positive response threshold of 100 sfu/million CD3^+^ T cells indicated (dotted line).

We wished to determine whether these novel HCMV CD8^+^ T cell IL-10 responses were established during primary infection or are a product of changes to the memory CD8^+^ T cell pool over time as the virus persists and periodically reactivates [51]. A number of studies of the development of the immune response in primary HCMV infection have been performed in adult settings [52,53,54]. However, in immunocompetent adults, primary HCMV infection is mild and often does not require medical intervention, making the identification of individuals with primary infection and the longitudinal analysis of immune responses difficult. A surrogate model for studying primary infection in adults utilizes solid organ transplantation, cases where a HCMV-seropositive organ is transplanted into a seronegative recipient (D^+^R^−^). A number of studies using such transplant patients as model for primary HCMV infection—as the time of infection can be determined post-transplant surgery—have been undertaken, which has allowed the kinetics of the developing immune response for both CD4^+^ and CD8^+^ T cells to be assessed [55,56]. In collaboration with AMC Amsterdam, a cohort of seven D^+^R^−^ kidney transplant patient samples (Table S2) from multiple timepoints post transplantation were analyzed using IFNγ and IL-10 FluoroSpots to establish when post-infection HCMV-specific T cells to a broad range of proteins develop. The IFNγ and IL-10 CD3^+^ T cell responses to stimulation with three different mixes of HCMV proteins, including IE1 and IE2 proteins (Figure 4A), latency-associated proteins UL138, US28, LUNA and vIL-10 (Figure 4B), and pp71 and US3 (Figure 4C) were analyzed. The results show that IFNγ responses (above the threshold of 100 sfu/million CD3^+^ T cells) to all three protein mixes were identifiable within the first 4 weeks post-transplant; however, only pp71/US3-peptide stimulation developed an IL-10 response which was maintained. The mixed latency-associated protein peptides only had a low frequency IL-10 response at discreet time points and this response was not maintained over the same time course. The generation of both IL-10 and IFNγ responses by these seven patients was not influenced by multiple incidents of viremia (e.g., Pt 136, Pt 352 and Pt 574) compared to a single peak of viremia early on post-transplant (e.g., Pt 197, Pt 365 and Pt 439). The individual patient responses to the three HCMV-protein stimulation conditions overlying their viremic episodes are shown in Figure S2.

**Figure 5:**
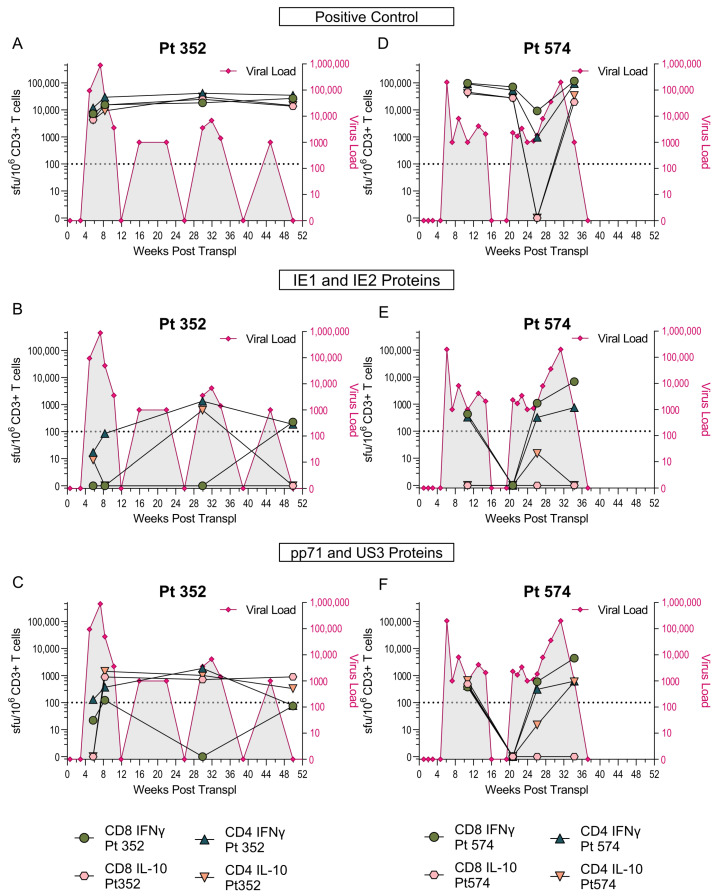
HCMV-specific IL-10 responses that arise during primary infection are produced by both CD8^+^ and CD4^+^ T cells. Four PBMC samples from D+R- patients 352 (**A**–**C**) and 574 (**D**–**F**) post-transplant time course were depleted of CD4^+^ or CD8^+^ T cells to assess which T cell subset is producing both IFNγ and IL-10 in response to HCMV-protein stimulation. The IFNγ and IL-10 CD8 (circle points) and CD4 (triangle points) T cell responses of both patients to the positive control (**A**,**D**), IE1 and IE2 proteins, (**B**,**E**) pp71 and US3 proteins (**C**,**F**) stimulation are shown, with the positive response threshold of 100 sfu/million CD3+ T cells indicated (dotted line). The virus load across the time course for each patient is shown (pink line).

As these assays were performed on total T cells, we wanted to determine whether the primary IL-10 responses are generated by CD8^+^ and/or CD4^+^ T cells. Therefore, CD4 and CD8 depletions (Figure S3) of PBMC samples from the time-course series of two transplant patients (Pt 352 and Pt 574) were performed, and these cells were then stimulated with an IE-protein mix or the pp71/US3 mix. The frequency of IFNγ and IL-10 CD4^+^ and CD8^+^ T cells was determined and correlated with the HCMV viral load (Figure 5). The results show that the IL-10 response to pp71/US3 was composed of both CD8^+^ and CD4^+^ T cells in both patients, however while Pt352 maintained the CD8 IL-10 response, over time the response in Pt574 was lost from peripheral blood (Figure 5C,F). The dynamics of the changing viral load for each patient are also shown on the graphs and the appearance of a CD4^+^ IL-10 response to the IE peptide in Pt352 at week 30 coincides with a viremic episode. An IL-10 response to IE1 and IE2 peptide stimulation was not seen at any of the other timepoints for Pt352 in this depletion experiment or the previous total CD3^+^ T cell experiment (Figure S2A). Taken together, the results suggests that while the IL-10 responses to pp71/US3 arise during the primary immune response, and in most individuals are maintained into long-term memory, the IL-10 responses specific for latency-associated proteins do not.

### 3.3. HCMV-Specific CD8^+^ IL-10-Secreting T Cells Are a Heterogeneous Population

**Figure 6:**
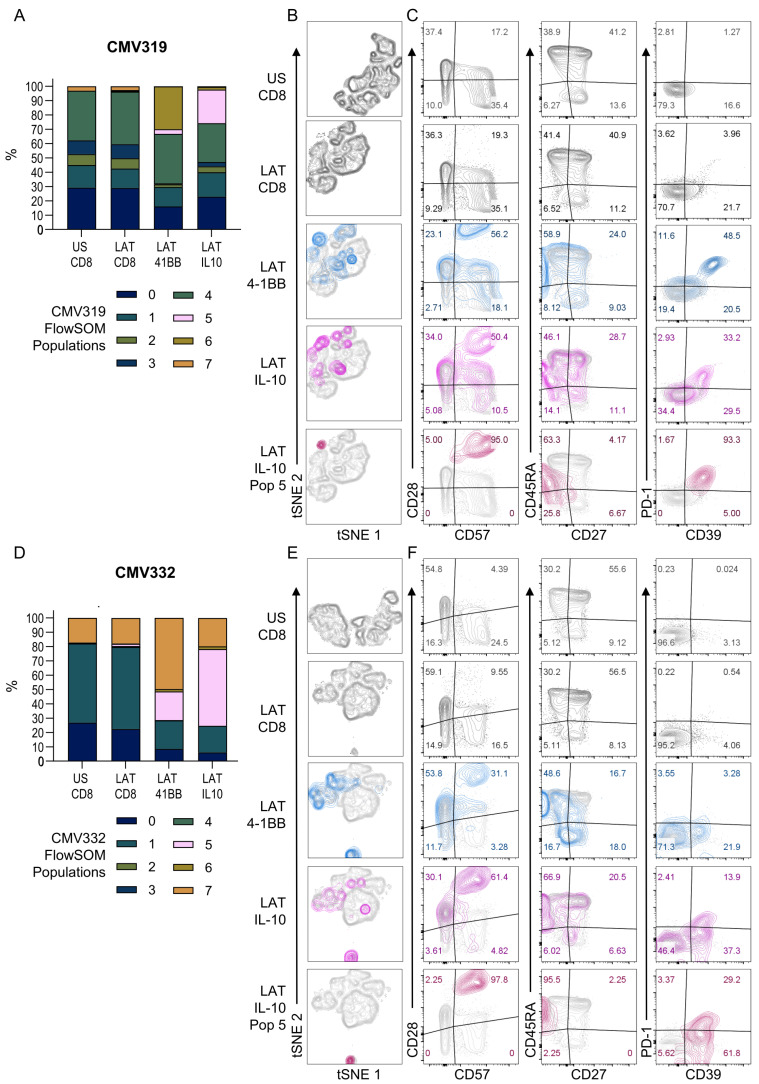
Characterization of HCMV latency-associated protein-specific CD8^+^ T cells secreting IL-10. PBMC were stimulated for 6 h with HCMV peptide pools and IL-10 secretion detected by the use of an IL-10-secretion assay kit. Additionally, HCMV-specific CD8^+^ T cell responses were identified by the upregulation of 4-1BB on the cell surface. The phenotype markers of T cell memory subsets and the differentiation status of CD27, CD45RA, CD28 and CD57 were included alongside PD-1 and CD39 markers associated with CD8^+^ IL-10-secreting T cells. Acquired data from each donor was analyzed using dimensionality reduction (tSNE, Flowjo 10.8.1) and clustering analysis (FlowSOM plugin, Flowjo). A summary of the FlowSOM population clustering distribution for unstimulated (US) total CD8s and latency associated protein stimulated (LAT) total CD8, 4-1BB-positive and IL-10-secreting cells is shown for donor CMV319 (**A**) and CMV332 (**D**). The dimensionality reduction tSNE plots are shown for total CD8s in both US and LAT conditions with the 4-1BB-expressing (blue overlay), IL-10-secreting (pink overlay) and IL-10 FlowSOM population 5 (purple overlay) also indicated (CMV319, **B** and CMV332, **E**). The T cell phenotype of the different populations are also shown with CD28 vs. CD57, CD45RA vs CD27 and PD-1 vs CD39 plots with donor-specific gating shown (CMV319, **C** and CMV332, **F**).

CD8^+^ T cells that secrete IL-10 have been described in the mouse and the rat and in some studies of human CD8^+^ T cell-memory populations [35,37,57]. The inhibitory receptor PD-1 [58] has been shown to be increased in T cell populations that have low or no IFNγ secretion and may secrete IL-10 in HCMV-specific CD4^+^ T cells [59]. Additionally, the cell surface enzyme CD39 has also been associated with CD8^+^ T regulatory cells [35,36]. We used a flow cytometry-based IL-10 capture assay in conjunction with activation (4-1BB) and T cell-memory subset and differentiation markers (CD27, CD45RA, CD28 and CD57) alongside PD-1 and CD39 as IL-10 and CD8^+^ T regulatory markers to determine the phenotype of HCMV-specific IL-10-secreting CD8^+^ T cells. The acquired data from each donor was analyzed using dimensionality reduction (tSNE) and clustering analysis (FlowSOM plugin) according to the workflow outlined in Figures S4 and S5. The clustering analysis performed on each donor independently revealed a similar IL-10-producing CD8^+^ T cell population in the latency-associated proteins peptide pool-stimulated cells (identified as population 5, light pink bars in Figure 6A,D). It was also apparent that the activated 4-1BB-positive populations and IL-10-secreting populations had a distinct phenotype from both the FlowSOM analysis (all eight populations mapped on the tSNE for both donors are summarized in Figure S6) and dimensionality reduction tSNE analysis shown in Figure 6B,E. Whilst the IL-10 response encompasses a number of populations, population 5 was the only one that was enriched in the IL-10-secreting HCMV-specific CD8^+^ T cells, rising from 0.01% and 0% in the unstimulated CD8 cells, to 23.4% and 53.6% in the latent stimulated IL-10-secreting cells for CMV319 and CMV332, respectively.

We observed an increase in PD-1 (CMV319, 4.1% to 60.1%; CMV332, 0.25% to 6.8%) and CD39 (CMV319, 17.9% to 69.0%; CMV332, 3.1% to 25.2%) expression on the activated HCMV-specific CD8^+^ T cells compared to resting. The combined proportion of HCMV-specific IL-10 cells enriched in population 5 co-expressing CD39 and PD-1 (CD39^+^PD-1^+^) differed between the two donors, ranging from 93.3% in donor CMV319 to 29.2% in donor CMV332. Interestingly, the differential expression of PD-1 and CD39 in the IL-10-positive population 5 was reflected in an experiment where ex vivo PD-1-expressing CD8^+^ T cells were depleted prior to stimulation with HCMV latency-associated protein mix in a dual IFNγ and IL-10 FluoroSpot assay (Figure S7). In this experiment, the depletion of resting CD8^+^ PD-1^+^ T cells from donor CMV319 resulted in a significant reduction in the frequency of IL-10-secreting cells, which was not seen in donor CMV332. A memory-T cell subset analysis of the HCMV-specific and HCMV-specific IL-10-secreting cells (Figure 6C,F), showed that the CMV-specific activated CD8^+^ T cells were a majority of TEMRA cells (CD27—CD45RA^+^) in both donors (CMV319, 58.9%; CMV332, 48.6%, shown in the blue overlay) and this phenotype was substantially increased in the IL-10 FlowSOM population 5 (burgundy overlay; CMV319, 63.3%; CMV332, 95.5%). In addition, most of the HCMV-specific cells were expressing CD28^+^CD57^+^ (CMV319, 95%; CMV332, 97.8%). The phenotype we observed for HCMV-specific IL-10-producing cells is distinct as it does not contain many CD28-negative cells, a phenotype associated with PD-1^+^ IL-10-secreting CD8^+^ T cells in HCV [40]. Overall, our results show that IL-10-secreting CD8^+^ T cells are CD28hi, CD27- CD45RA^+^ and CD39^+^ with a heterogenous expression of PD-1.

### 3.4. Diverse HCMV-Specific IL-10 CD8^+^ T Cells May Be Tissue-Resident

Studies of immune responses in humans often rely on the peripheral blood compartment as it generally allows harmless and relatively easy access to immune cells. However, peripheral blood only comprises 2% of the total lymphocyte population present in the body [60]. In our previous analysis of HCMV-specific CD8^+^ T cells’ IFNγ responses we observed that the frequency and diversity of these responses were not significantly changed over time; although there were fluctuations in the magnitude of the responses made by individual donors, they maintained the positive response to the HCMV proteins [13]. In the course of our experiments investigating HCMV-specific IL-10 responses with repeated samples from the same donors it was apparent that the frequency of CD8^+^ T cell IL-10 responses in the peripheral blood fluctuates considerably over time. In four separate donors, the IL-10 responses to the proteins pp65, UL138, LUNA, US28 and vIL-10 change in magnitude or are negative and sometimes undetectable at different timepoints (Figure S8A).

The changes in the antigen specificity of the CD8^+^ T cell IL-10 profile in the peripheral blood suggested that responses may be more tissue-resident in nature. A major drawback of studying human tissue sites is that they tend to only be accessible, e.g., during medical procedures where, often, the individual may be unwell, thereby affecting the composition of the tissue-resident immune compartment. Bone marrow-derived mononuclear cells (paired with peripheral blood from the same donor) do enable the analysis of a tissue site that is also enriched in the proportion of lymphocytes present; bone marrow can contain up to 10% of lymphocytes, compared to 2% in peripheral blood [60]. In a preliminary investigation analyzing the CD4^+^ and CD8^+^ T cell responses to 10 HCMV proteins in paired bone marrow (BM) and peripheral blood (PB) from a HCMV-seropositive donor we observed positive IL-10 responses to the proteins US28, IE1 and IE2 in the bone marrow which were absent from the peripheral blood (Figure S8B). The enrichment in diversity and numbers of IL-10-secreting CD8^+^ T cells in the bone marrow is of interest as CD34^+^ bone marrow progenitor cells are a known site of latent HCMV carriage [6], and this could mean that HCMV-specific IL-10-secreting CD8^+^ T cells may play a role in the maintenance of HCMV latency.

## 4. Discussion

It is well established that CD8^+^ T cells specific to HCMV play an important role in mitigating the detrimental effects of both primary infection and reactivation of the virus [9]. There has been extensive characterization of the HCMV-specific CD8^+^ T cell response to HCMV infection but few studies have addressed whether the secretion of IL-10 also occurs. The few reports of HCMV-specific IL-10 CD8^+^ T cell or regulatory responses have been in patients with other infections such as HIV [61] and HBV [42], or in liver transplant patients who have progressed to CMV disease [41], or in ageing studies using a pp65-specific tetramer [62]. There are also examples in the mouse model of cytomegalovirus (MCMV) of IL-10 production by CD8^+^ T cells; they have been identified in the salivary gland and in liver-infiltrating lymphocytes using both cytokine staining and IL-10 reporter mice [63,64,65,66]. We have shown here that there are widespread CD8^+^ T cell IL-10 responses to a number of different HCMV proteins and that a majority of the donors examined produce an IL-10 response to one or more HCMV proteins. By surveying the CD8^+^ T cell responses to 10 different HCMV proteins we were able to determine that the latency-associated proteins (US28, UL138, LUNA, vIL-10), the immune evasion protein US3 and the late structural protein pp71 often stimulated a dominant IL-10 response in our donor cohort. We also observed that the production of IL-10 was rarely seen from cells that were also producing IFNγ in response to HCMV stimulation, which is unusual as dual IL-10 and IFNγ-producing CD8^+^ T cells are often described in other virus infections [61,67,68,69,70]. Single IL-10-producing T cells have been observed in chronic HIV and HCV infections [38,61,70,71,72], suggesting that a persistent viral load may bias responses towards a single IL-10-producing CD8^+^ T cell population.

The analyses of IL-10-secreting T cells have tended to concentrate on CD4^+^ T cells as CD8^+^ regulatory T cells are not as frequently observed as their CD4^+^ counterparts [33]. Studies that have been undertaken to characterize and phenotype IL-10-producing memory CD8^+^ T cells have revealed a heterogeneous population with distinct differences in the phenotypes observed in rodent models and human immune responses [34,35]. The expression or the loss of expression of certain cell surface molecules on CD8^+^ memory T cells have been identified in humans as associated with immunomodulatory effects. The major phenotypes identified in infectious diseases include CD28-negative CD39-positive CD8^+^ T cells in HIV infection [36], and the upregulation of the inhibitory receptor PD-1 [58] on IL-10-secreting cells has also been observed in HIV, HBV and HCV [38,39,40]. The expression of PD-1 in CMV-specific CD4^+^ T cells has also been shown to suppress the production of anti-viral cytokines [59], and CMV-specific CD8^+^ T cells in HBV patients had increased PD-1 which correlated with the loss of IFNγ [42]. The assessment of the phenotype of CMV-specific IL-10-secreting CD8^+^ T cells in our study revealed an interesting and somewhat unique phenotype of an effector memory population expressing CD45RA which had a CD28^+^ CD39^+^ phenotype—this contrasts with other reports described for HIV [36]. The HCMV-specific IL-10-positive cells were also mainly CD28^+^ CD57^+^, a mid-differentiation CD8^+^ T cell memory population which has been shown to have increased apoptosis and co-inhibitory and chemokine receptors [73]. The expression of PD-1 by the IL-10-producing CD8^+^ T cells to the same HCMV proteins varied between 90% and 30% in the two donors examined and this difference in expression was confirmed by our experiments involving the ex vivo depletion of PD-1. It is interesting to note that, in the murine model of CMV infection, the expression of PD-1 was high in tissue-resident memory T cells in the salivary gland and maintained over time, whereas spleen and kidney-localized T cells lost PD-1 expression [74]. This suggests that the between-donor variation in PD-1 expression on IL-10-secreting CD8^+^ T cells observed here may be due to the development of the HCMV-specific T cell response in an individual donor.

Our analysis of when during HCMV infection IL-10 CD8^+^ T cells arise revealed distinct differences in the HCMV proteins generating an IL-10 response in CD8^+^ T cells present in the peripheral blood. In seven primary HCMV infections in D^+^R^−^ kidney transplant patients we measured IFNγ responses from as early as 4 weeks after infection to mixes of HCMV proteins containing IE1 and IE2, latency-associated proteins (UL138, US28, LUNA and vIL-10), pp71 and US3. In contrast, only the mix of pp71 and US3 proteins generated IL-10 responses in these donors, which shows that these responses were detectable from early time points post infection and are maintained in the long-term memory responses. These results raise the question as to when IL-10 responses to the latency-associated proteins arise. Are they generated as a result of longer-term viral carriage than the timepoints studied here, which extended to 3 years in one donor but was 2 years or less for the remaining six donors? It is possible that the memory IL-10 T cell responses to latency-associated proteins arise due to the conversion of the existing IFNγ-secreting specific CD8^+^ T cells following restimulation during periodic episodes of reactivation. Alternatively, it may be that the latency-associated protein-specific CD8^+^ T cells are generated at tissue sites of infection and are not visible in the peripheral blood at these early time-points. There is evidence from the murine model of cytomegalovirus infection that CMV-specific CD8^+^ T cells are present at tissue sites, e.g., the salivary gland, brain and lungs, where they play an important role in controlling viral infection [75]. From our studies, we have evidence that the presence of HCMV-specific IL-10-secreting CD8^+^ T cells in the peripheral blood fluctuates over time, and in our analysis of bone marrow-derived mononuclear cells we saw IL-10 responses to all the HCMV proteins tested, though some of these responses were absent from the PBMC. The bone marrow is an important tissue site in the etiology of HCMV infection as it is a known resident site of CD34^+^ cells which have been shown to carry the latent virus in natural infection [76]. We have previously hypothesized that there is a suppressive microenvironment around latently infected CD34^+^ cells due to the presence of latency-induced secretion of cIL-10 and TGFβ by latently infected CD34^+^ cells, and also due to the recruitment of cIL-10-secreting T cells specific for latency-associated proteins [21]. The effect of this microenvironment, enriched for cIL-10 and vIL-10 produced by the latent virus infection [77], would likely be a reduction in the recognition and clearance of HCMV-latent infected cells by anti-viral cytotoxic and cytokine-producing T cells, helping the maintenance of the latent infection.

It is clear from the work presented here that the HCMV-specific IL-10-secreting CD8^+^ T cell is a particular feature of the HCMV-specific CD8^+^ T cell repertoire and it potentially plays an important role in modulating the HCMV-specific immune response in tissue sites. The characterization and phenotyping of these cells requires further work, particularly due to the complex heterogeneity of CD8 IL-10-secreting and regulatory cells shown here and in other publications [34]. It is also apparent that investigating the immune populations present at tissue sites such as bone marrow (along with the peripheral blood) will be essential in deciphering the full repertoire of the HCMV-specific T cell response. This will allow us to answer questions such as whether tissue-resident T cell populations are derived from the same populations present in peripheral blood or whether there are distinct T cell lineages in different immune compartments. The employment of single-cell population analyses (e.g., single-cell RNA-seq and high dimensional flow cytometry) and, importantly from a T cell perspective, T cell receptor (TCR) sequencing [78], will be required to understand the dynamics of these populations and whether the TCR composition of the same HCMV peptide-specific CD8^+^ T cells varies across tissue sites or cytokine-secreting phenotypes. Future work will also need to answer whether the IL-10 and IFNγ responses observed in response to HCMV protein peptide pool stimulation are to the same or different antigenic peptide sequences. Addressing this question will require peptide mapping of the latency-associated proteins and also US3 and pp71, as previous work has concentrated on the investigation and mapping of the immunogenic responses present in pp65 and IE1 proteins. The other drawback of existing reagents to specific peptide sequences, such as the very well characterized and used pp65 HLA A2-restricted peptide, is the reliance on certain HLA alleles that are more commonly expressed in European populations [79]. The single-cell characterization of different HCMV-specific CD8^+^ T cell responses will also require the mapping of individual donor responses to specific peptides as the functional diversity we observe at the protein level may be due to different epitopes being recognized.

Improving our understanding of the full functionality of HCMV-specific T cells present at multiple sites in the human host will undoubtedly help to inform future therapeutic strategies for HCMV. HCMV reactivation is also a significant cause of morbidity and mortality in solid organ and hematopoietic stem cell transplantation [80] and in other immunosuppressed patients, such as those with severe sepsis [81], and recently intensive care patients with COVID-19 [82]. The importance of suppressive HCMV-specific CD8^+^ T cells in these disease settings has been shown in severe sepsis patients where the increased frequency of PD-1^+^ CD8^+^ T cells is associated with HCMV disease [19] and the loss of polyfunctional CD8^+^ T cell responses. Currently, in the field of solid organ transplantation, the prevention and management of HCMV disease is an important clinical consideration and measuring HCMV-specific cellular immunity is currently employed as a marker of the potential for viremic episodes [18]. Improving our understanding of the dynamics of HCMV disease and relieving the need for prophylaxis treatment of viremia will require the assessment of the full repertoire of the CD8^+^ and CD4^+^ HCMV-specific T cell responses in patients. Understanding the HCMV-specific T cell response will also be necessary to prevent the rejection of the transplanted organ [83], as patients with an overabundance of the HCMV-specific IL-10 CD8^+^ T cell response may suppress the effective anti-viral T cell responses which may control HCMV disease in the absence of anti-viral drug regimens. Therefore, finding treatments for HCMV disease by understanding the complete nature of the immune response to the virus will help to improve clinical outcomes in many different areas.

## Data Availability

The data presented in this study are available on request from the corresponding author.

## Supplementary Materials

The following supplementary material can be downloaded at: https://www.mdpi.com/article/10.3390/pathogens11121530/s1. It includes Supplementary Tables S1: ARIA cohort subset characteristics, S2: AMC Primary patient’s details, S3: Antibody clones and manufacturer information, S4: Summarized HCMV protein IL-10 responses and Supplementary Figures S1: Gating strategy and representative unstimulated positive control and pp65 stimulated cells for ICS, S2: AMC Patients individual Latency associated, IE1/IE2 and pp71/US3 IFNγ & IL-10 responses with virus load and seroconversion data, S3: Purity of CD8 and CD4 T cell depletion of two AMC patients, S4: Gating strategy and Processing Workflow of IL-10 phenotype analysis, S5: CMV319 and CMV332 pre-processing activation, IL-10 capture and T cell memory, differentiation and regulatory phenotype for unstimulated and latency associated protein stimulation, S6: FlowSOM Population tSEN overlays showing distribution, S7: Depletion of PD-1 from resting CD8*+* T cells results in varied production of IL-10 in response to HCMV peptide stimulation, S8: Fluctuation of IL-10 CD8*+* T cell HCMV specific responses in peripheral blood and identification of CD8*+* T cell IL-10 responses in the Bone Marrow.
