# Supplementary material for: IL-10-Secreting CD8+ T Cells Specific for Human Cytomegalovirus (HCMV): Generation, Maintenance and Phenotype
Source: Pathogens. 2022 Dec 13;11(12):1530. doi: 10.3390/pathogens11121530 (PMC9781655; doi:10.3390/pathogens11121530)
Supplement: Supplementary file 1 [file pathogens-11-01530-s001.zip › pathogens-2065060-supplementary.pdf]

Jackson S.E. et al 2022 Supplementary Material

1 Supplementary Tables

Table S1: ARIA cohort subset characteristics

Donor Characteristics									
Variable (Mean ± S.D.)		All Ages		Young (< 40 years)		Middle (41 - 64 years)		Old (> 65 years)	
		CMV+ve	CMV-ve	CMV+ve	CMV-ve	CMV+ve	CMV-ve	CMV+ve	CMV-ve
Donors (n)		46	3	17	1	12	0	17	2
Age		53.7 ± 16.5	58.0 ± 15.0	34.8 ± 4.9	37.0 ± 0.0	54.9 ± 5.6	-	71.7 ± 2.9	68.5 ± 2.5
Male (M) %		54%	33%	47%	0%	67%	-	53%	50%
CMV IgG (ISR)	All	3.62 ± 1.13	0.26 ± 0.14	3.51 ± 1.09	-	3.46 ± 0.91	-	3.83 ± 1.30	-
	M	3.53 ± 0.90	-	3.02 ± 0.69	-	3.79 ± 0.66	-	3.76 ± 1.04	-
	F	3.72 ± 1.35	-	3.94 ± 1.19	-	2.79 ± 0.96	-	3.92 ± 1.48	-

Summary of the number of donors and age ranges, % male and serum HCMV IgG levels [Immune status ratio (ISR)]

Table S2: AMC Primary patient's details

**Patient ID**	**Age at Tx**	**Immuno-suppressive therapy**	**Proph-ylaxis**	**Antiviral therapy**	**First detection of virus (week post Tx)**	**First peak viraemia (week post Tx)**	**CMV IgG+ (weeks post Tx)**	**Last sample (week post tx)**
133	66	P/CsA/MMF	No	No	8.7	12.14	15.71	123.3
136	31	P/CsA/MMF	No	Yes	6.6	8.57	10.57	78.6
197	31	P/MMF/FK/Cd25mAb	No	No	14.0	14.00	14.00	157.9
352	26	P/CsA/CD25mAb/MMF	No	Yes	4.9	7.14	7.14	97.4
365	54	P/CsA/CD25mAb/MMF	No	Yes	6.9	6.86	8.57	116.7
439	21	P/MMF/FK/Cd25mAb	No	Yes	6.6	9.00	10.00	16.6
574	50	P/MMF/FK/Cd25mAb	Months 1-3, 6-9	No	6.1	6.29	25.43	37.3

Immunosuppressives key:
P – Prednisone; CsA – Cyclosporin; FK – Tacrolimus; MMF – mycophenolate mofetil.

Table S3: Antibody clones and manufacturer information

Antigen	Fluorochrome	Clone	Isotype	Cat. No.	Supplier	Experiment
CD3	FITC	UCHT1	IgG1κ	300406	BioLegend	Purity (CD4/8 Depl)
CD4	PE	RPA-T4	IgG1κ	300508	BioLegend	Purity (CD4/8 Depl)
CD8	PerCP-Cy5.5	RPA-T8	IgG1κ	301032	BioLegend	Purity (CD4/8 Depl)
CD14	BV510	M5E2	IgG2aκ	301842	BioLegend	Dump channel
CD19	BV510	HIB19	IgG1κ	302242	BioLegend	Dump channel
CD3	BV650	OKT3	IgG2aκ	317324	BioLegend	ICS & IL10 Capture
CD4	BV605	OKT4	IgG2bκ	317438	BioLegend	ICS & IL10 Capture
CD8	BV570	RPA-T8	IgG1κ	301038	BioLegend	ICS & IL10 Capture
CD69	Pacific Blue	FN50	IgG1κ	310920	BioLegend	ICS
4-1BB	PE-Cy5	4B4-1	IgG1κ	309808	BioLegend	ICS & IL10 Capture
IL-10	PE	JES3-9D7	Rat IgG1κ	501404	BioLegend	ICS
IFNγ	BV786	4S.B3	IgG1κ	563731	BD Biosciences	ICS
PD-1	FITC	EH12.2H7	IgG1κ	329904	BioLegend	PD-1 depletion
CD4	FITC	RPA-T4	IgG1κ	300506	BioLegend	Purity (PD-1 Depl)
CD8	PE	RPA-T8	IgG1κ	301008	BioLegend	Purity (PD-1 Depl)
CD3	PerCP-Cy5.5	OKT3	IgG2aκ	317336	BioLegend	Purity (PD-1 Depl)
IL-10	PE	-	IgG1κ	130-090-434	Miltenyi Biotec	IL10 Capture
PD-1	BV421	EH12.2H7	IgG1κ	329920	BioLegend	IL10 Capture
CD57	PE-Dz 594	HNK-1	IgM κ	359620	BioLegend	IL10 Capture
CD40L	PerCP-Cy5.5	24-31	IgG1κ	310834	BioLegend	IL10 Capture
CD45RA	PE-Cy7	HI100	IgG2bκ	304126	BioLegend	IL10 Capture
CD27	APC	O323	IgG1κ	302810	BioLegend	IL10 Capture
CD28	AxF700	CD28.2	IgG1κ	302920	BioLegend	IL10 Capture
CD39	BUV737	Tu66	IgG2bκ	612852	BD Biosciences	IL10 Capture

Abbrv: BV = Brilliant Violet; BUV = Brilliant UV; Dz = Dazzle; AxF = Alexa Fluor; Depl = depletion.

Table S4: Summarized HCMV protein IL-10 responses

Protein	Responders	Percent Positive	Positive sfu/million Geomean (95% Confidence Intervals)
UL138	11/46	23.9	318.4 (196.7, 515.1)
US28	25/46	54.3	286.7 (213.3, 385.3)
LUNA	17/46	37.0	266.9 (204.9, 347.6)
UL144	15/46	32.6	240.6 (190.0, 260.1)
vIL-10	12/46	26.1	202.1 (157.0, 260.1)
pp71	28/46	60.9	440.1 (338.5, 572.0)
US3	24/46	52.2	314.8 (237.6, 416.9)
pp65	16/46	34.8	318.9 (230.1, 442.1)
IE2	17/46	37.0	199.8 (164.3, 243.0)
IE1	4/46	8.7	299.4 (81.14, 1105)

Number and percentage of IL-10 responding donors to each HCMV protein stimulation and the mean positive response to each protein (magnitude) in sfu/million.

2 Supplementary Figures

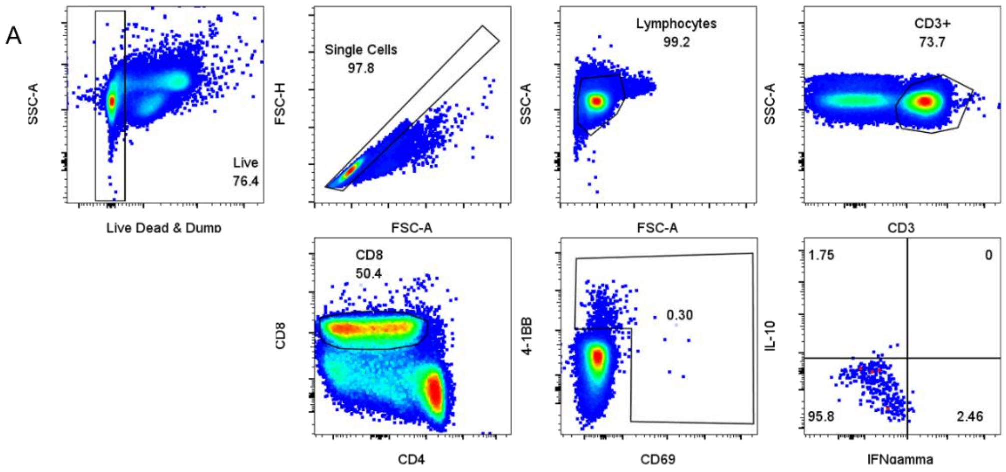

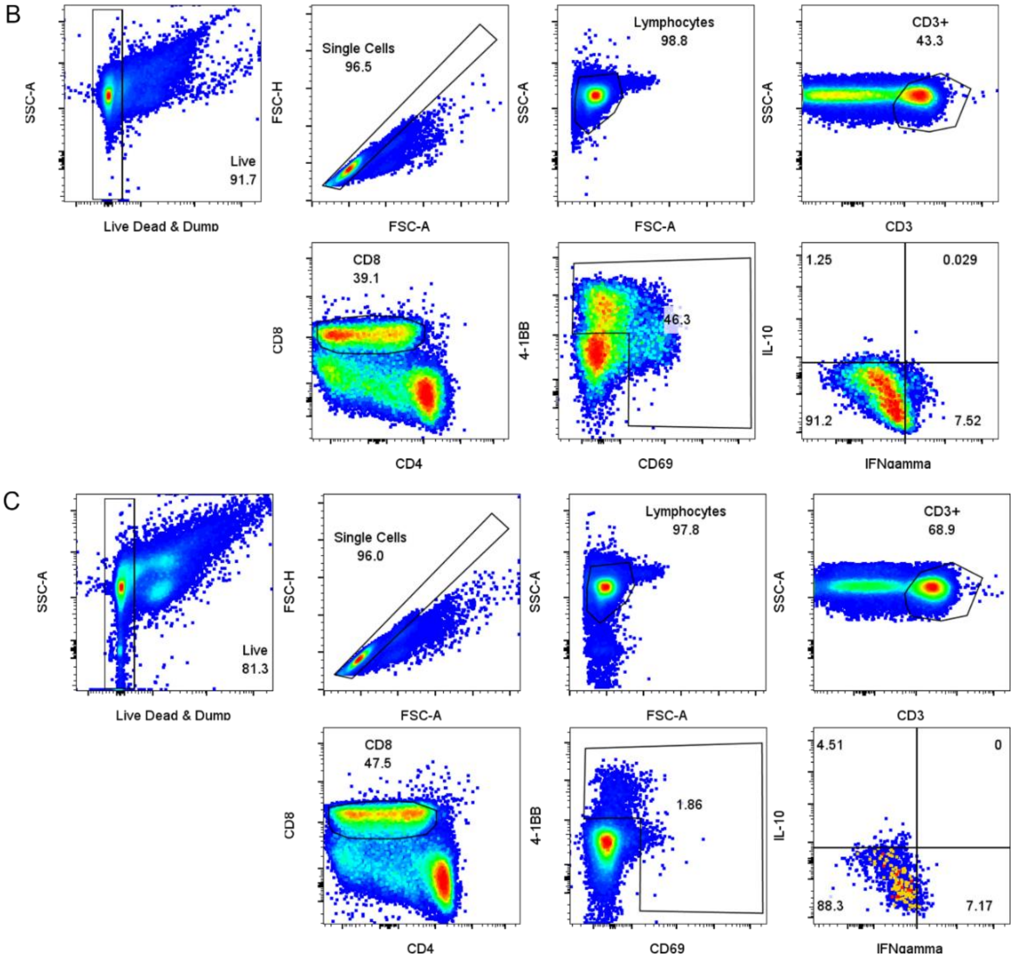

Figure S1. Gating strategy and representative Unstimulated, positive control and pp65 stimulated cells for ICS. PBMC were stimulated overnight with HCMV peptide pools in the presence of monensin. The gating strategy for defining the CD8+ T cells which upregulated the activation markers 4-1BB and CD69 and the cytokine production for the unstimulated (A), positive control (B) and pp65 stimulated (C) samples are shown. Briefly, live CD14 and CD19 negative cells were gated, then doublets excluded, a lymphocyte gate followed by CD3+ cells were drawn with CD8+ T cells then identified. The upregulation of 4-1BB and CD69 following HCMV stimulation is clearly shown by the percentage increase from 0.30% in the unstimulated (A) to 46.3% in the positive control (B) to 1.86% in the pp65 stimulated (C).

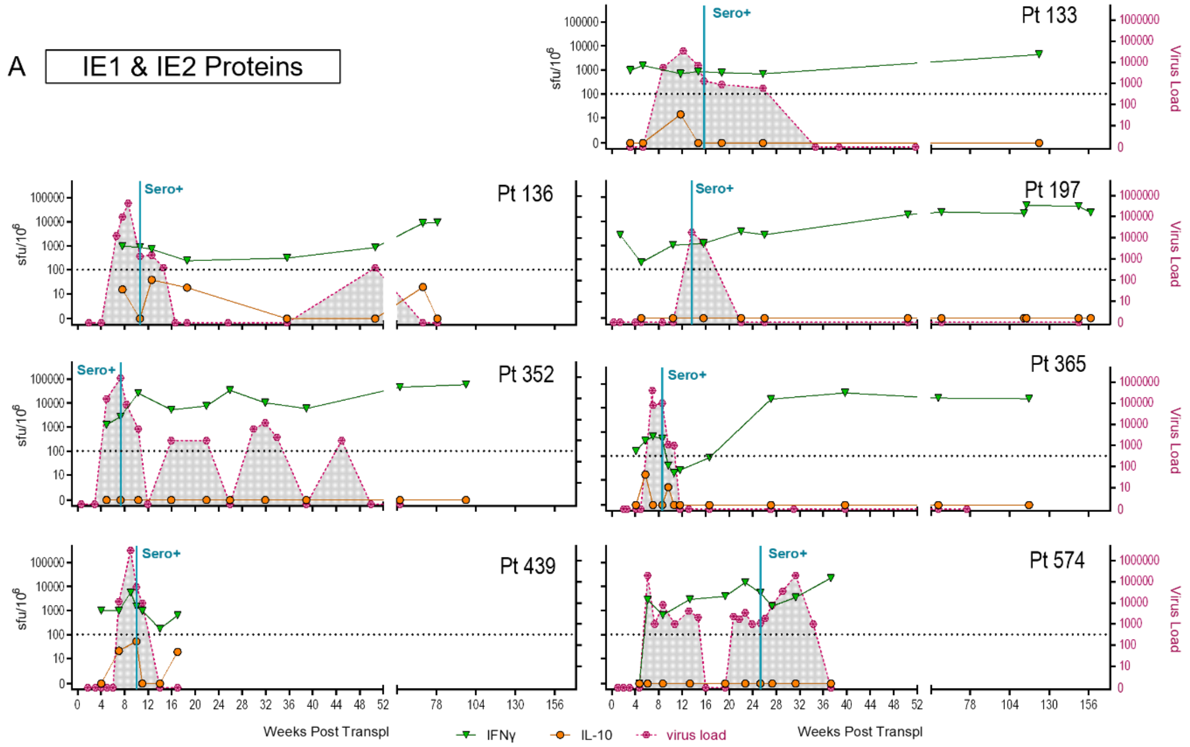

A
IE1 & IE2 Proteins
Pt 133
Pt 136
Pt 197
Pt 352
Pt 365
Pt 439
Pt 574
Sero+
sfu/10⁶
Virus Load
Weeks Post Transpl
IFNγ
IL-10
virus load

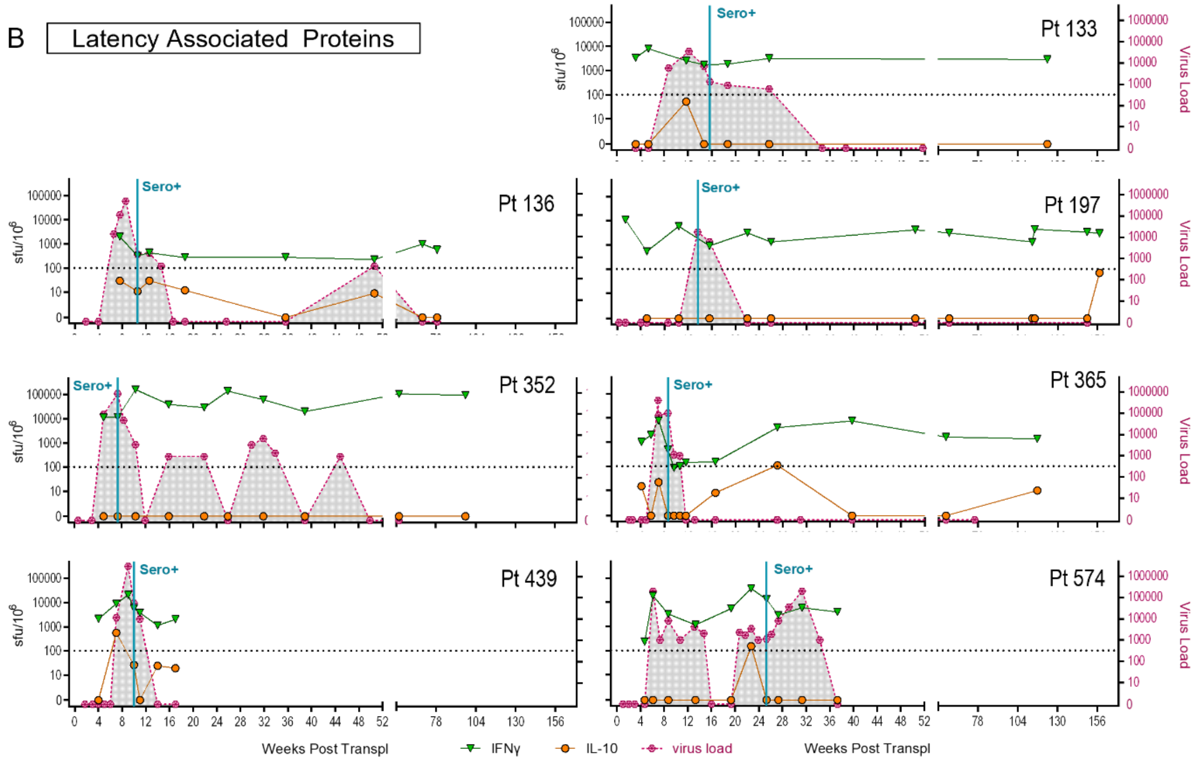

B
Latency Associated Proteins
Pt 133
Pt 136
Pt 197
Pt 352
Pt 365
Pt 439
Pt 574
Sero+
sfu/10⁶
Virus Load
Weeks Post Transpl
IFNγ
IL-10
virus load

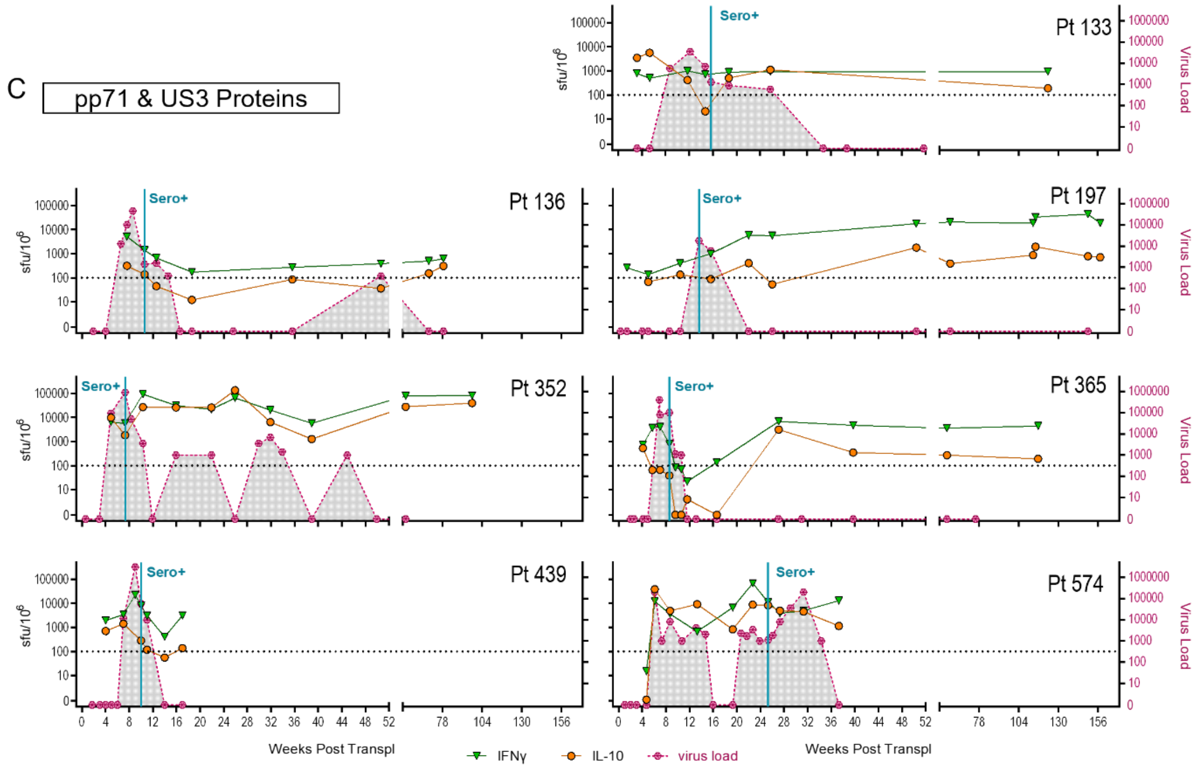

Figure S2. AMC Patients individual Latency associated, IE1/IE2 and pp71/US3 IFNγ & IL-10 responses with virus load and seroconversion data. The individual results for the seven D+R- kidney patients with primary HCMV infection are shown for IE1 and IE2 proteins (A), Latency associated proteins [UL138, US28, LUNA & vIL-10] (B) and pp71 and US3 proteins (C). T cell responses (spot forming units per 10^6 CD3+ T cells) were measured by IFNγ (green triangles connected by a solid line) and IL-10 FluoroSpot (orange circles connected by a solid line) with Virus load (copies/ml blood) (pink hexagons connected by a dashed line and shaded) and seroconversion of antibody response (turquoise line) also shown.

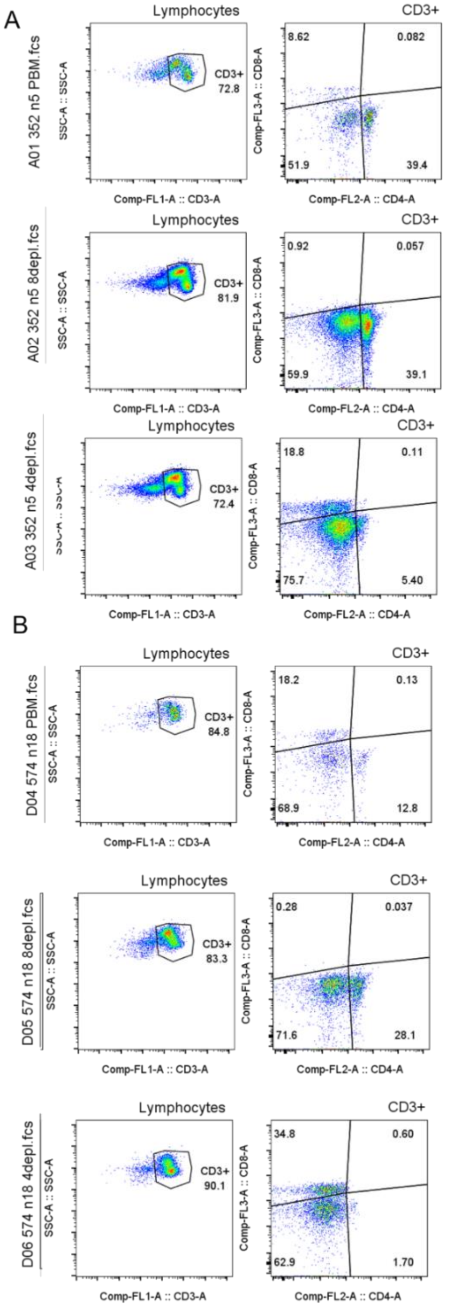

Patient 352						
Timecourse Sample	PBMC		CD8 depl		CD4 depl	
	CD4 %	CD8 %	CD4 %	CD8 %	CD4 %	CD8 %
n5	39.4	8.62	39.1	0.92	5.4	18.8
n7	48.5	8.03	53.0	0.71	5.5	15.4
n14	36.8	9.47	37.8	0.40	6.6	17.7
n18	41.5	21.1	55.7	0.61	3.9	38.3

Patient 574						
Timecourse Sample	PBMC		CD8 depl		CD4 depl	
	CD4 %	CD8 %	CD4 %	CD8 %	CD4 %	CD8 %
n7	31.3	13.4	47.8	0.89	6.99	18.3
n11	18.0	17.6	18.7	0.21	7.02	13.2
n15	8.45	3.22	12.2	0.18	6.00	6.49
n18	12.8	18.2	28.1	0.28	1.70	34.8

Figure S3. Purity of CD8 and CD4 T cell depletion of two AMC patients. Flow cytometry dot plots showing the proportion of CD3+ T cells and the CD4+ and CD8+ T cell distribution of patient 352 (A) and patient 574 (B) for one longitudinal sample of total PBMC, CD4 and CD8 depleted samples are shown. The tables summarize the percentage CD4+ and CD8+ T cells remaining in the depleted samples for all 4 longitudinal samples analyzed.

A

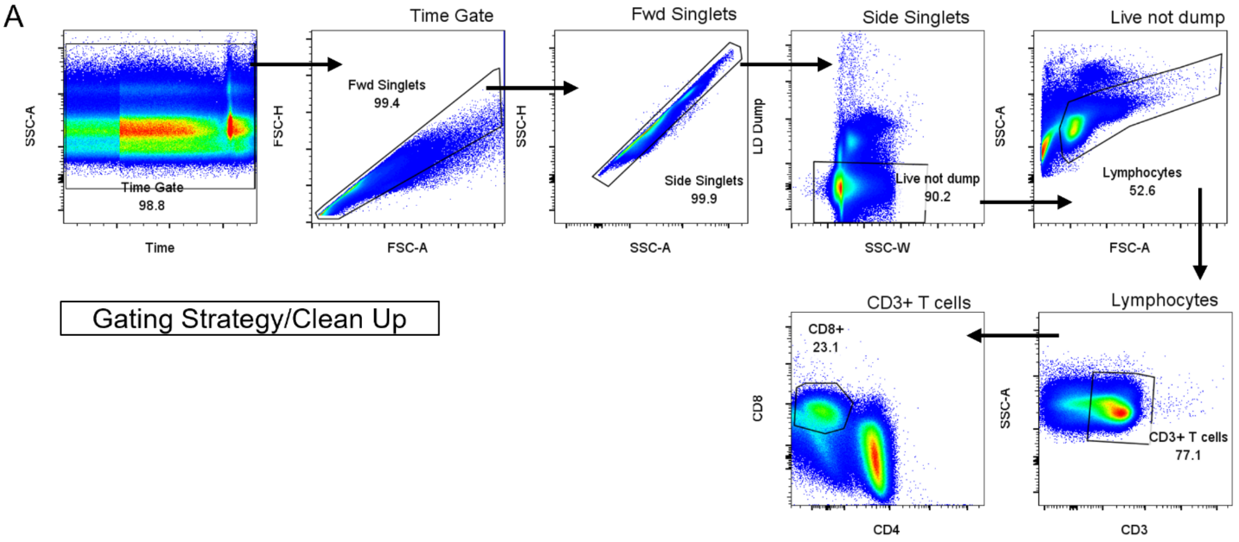

Gating Strategy/Clean Up

B

Dimension Reduction/Clustering Workflow

PRE-PROCESSING		
**Clean Up**	**Transformation**	**Concatenate**
• Compensation<br>• Gating to clean up data (A)	• Adjust scale of markers to visualize positive populations<br>• Derive gating for phenotype markers	• Combine gated CD8+ Unstimulated and Latent stimulated samples in to one file for processing.
Processed using FlowJo v10.8 Built in processes		

DIMENSIONALITY REDUCTION	CLUSTERING
**Visualisation**	**Transformation**
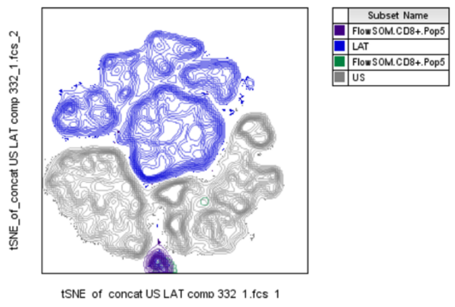	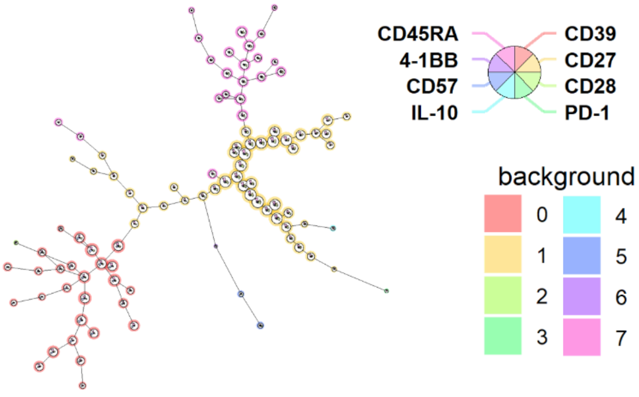
Used FlowJo v10.8 built in tSNE	Using FlowSOM Plugin

Figure S4. Gating strategy and Processing Workflow of IL-10 phenotype analysis. Responding CD8+ T cells were pre-analyzed to clean up the data for dimensionality reduction and clustering analysis. Shown (A) is the gating strategy used for analyzing these sample using FlowJo v 10.8.1 with representative plots from 1

donor sample. First a Time vs Side scatter gate was drawn, to identify the main flow of cells, then these cells were gated for forward scatter single cells (Forward scatter area (FSC-A) vs Forward scatter height (FSC-H)), side scatter single cells (Side scatter area (SSC-A) vs Side scatter height (SSC-H)), live not dump (CD14- CD19-) cells (SSC-W vs Live Dead Aqua dye, CD14 BV510 & CD19 BV510), then lymphocytes were gated (FSC-A vs SSC-A (log scale)). CD3 positive cells were identified (CD3 BV650 vs SSC-A) and then CD8 positive cells gated (CD4 BV605 vs CD8 BV570). Summarized in (B) is the workflow processes employed for analysis of the samples, indicating the pre-processing steps (clean-up, transformation and concatenation and combination of the CD8+ gated samples) followed by the visualization (employing t-SNE FlowJo v10.8.1) and transformation analysis of the data using the FlowSOM plugin within FlowJo v10.8.1.

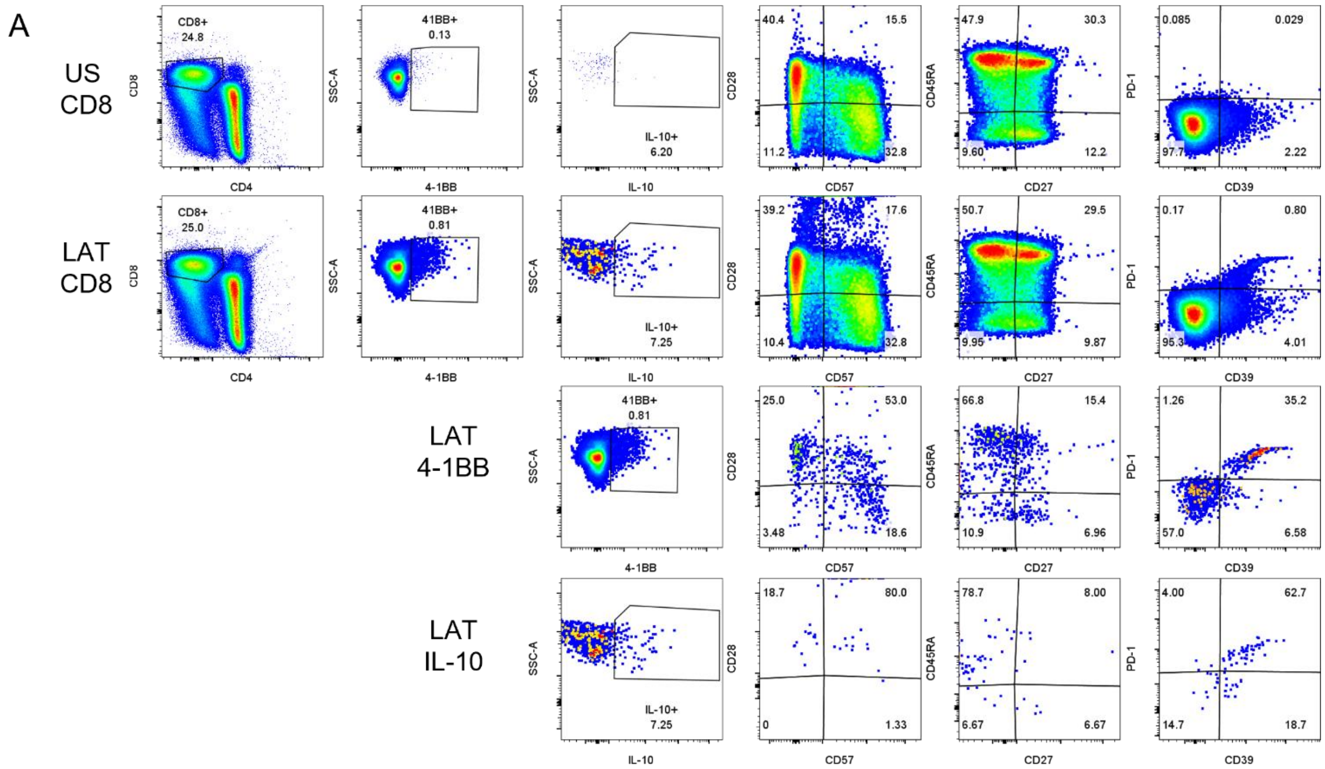

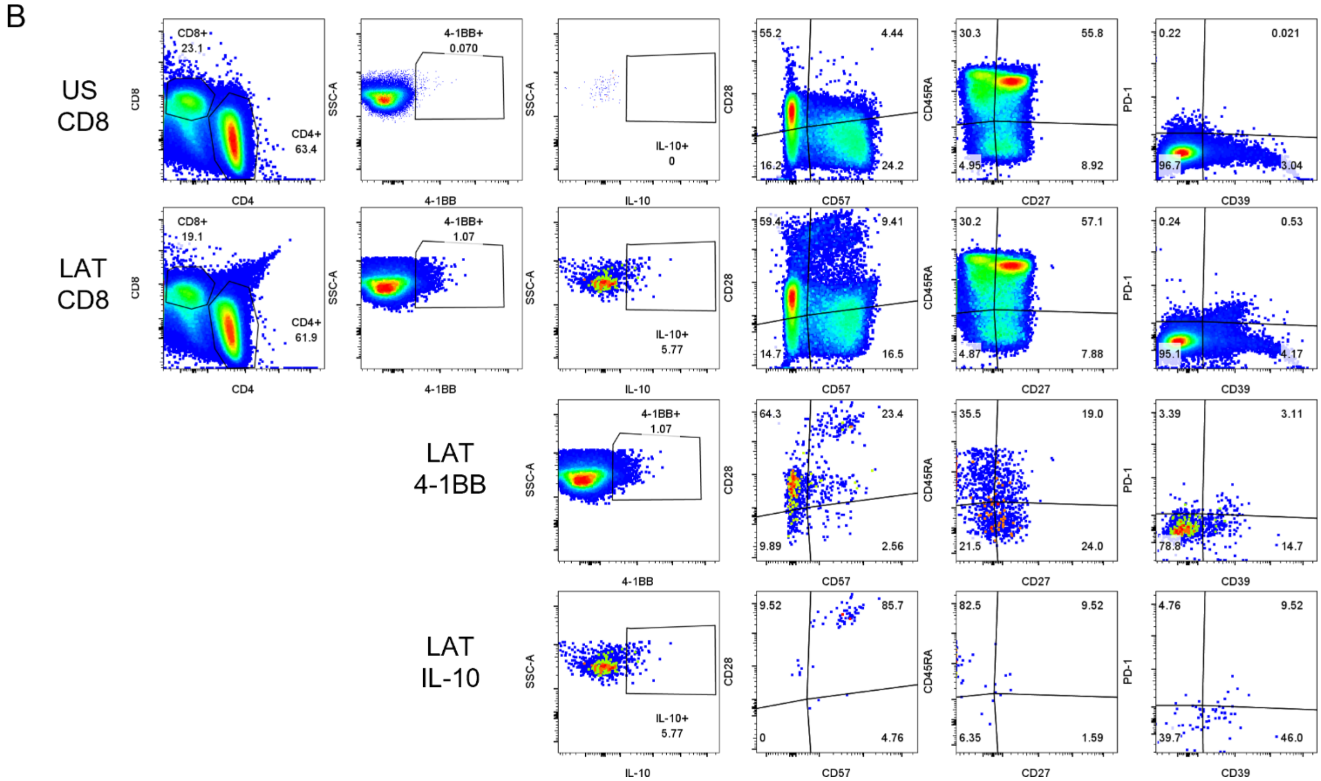

Figure S5. CMV319 and CMV332 pre-processing activation, IL-10 capture and T cell memory, differentiation and regulatory phenotype for unstimulated (US) and latency associated protein stimulation (LAT). T cell 4-1BB+, IL-10 capture staining and T cell phenotype analysis of the compensated and transformed unstimulated (US) and latency associated proteins stimulated (LAT) samples prior to concatenation, downsampling and high dimensionality processing (tSNE and FlowSOM) of donors CMV319 (A) and CMV332 (B) are shown. CD3 positive cells were identified and then CD8 positive cells gated (CD4 BV605 vs CD8 BV570), The 4-1BB expression (4-1BB PE-Cy5 vs SSC-A) of the CD8+ T cells is shown with a background expression of 0.13% & 0.07% in the unstimulated and 0.81% & 1.07% in the LAT stimulation for CMV319 (A) and CMV332 (B)respectively and then IL-10 upregulation (IL-10 PE vs SSC-A) of the 4-1BB positive cells, showing low background production in the unstimulated sample and increased IL-10 expression of 7.25% (A) & 5.77% (B) in the LAT stimulated cells. The CD8+ T cell differentiation phenotype of CD28 vs CD57, memory subsets of CD45RA vs CD27 and the CD8+ IL-10 regulatory cell phenotype markers of PD-1 vs CD39 of the total CD8+ T cell populations for US and LAT samples and the 4-1BB and IL-10 secreting populations of the LAT sample with individual donor gating reflecting the populations observed. The same gates are applied to the high dimensionality analysis samples in main Figure 5.

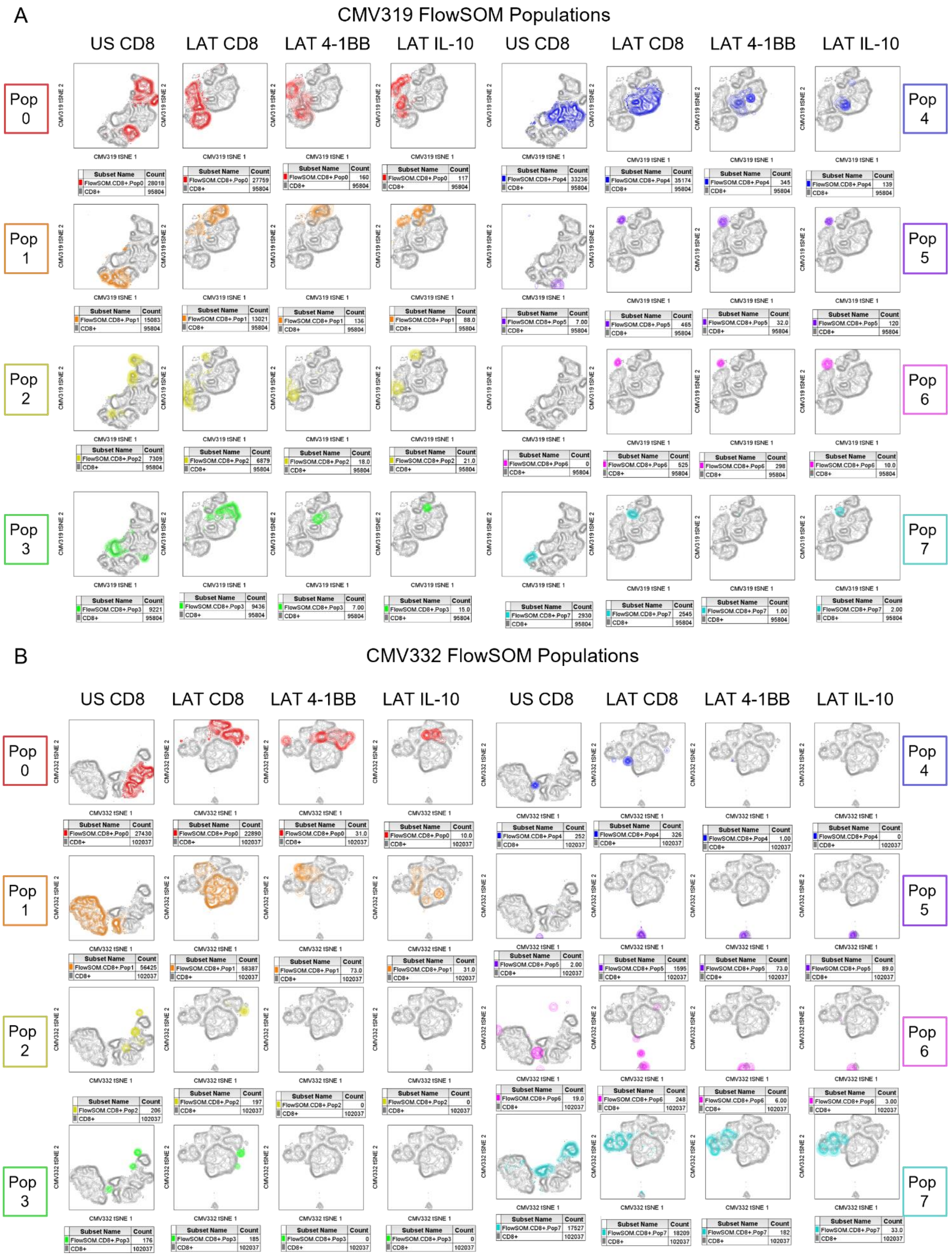
A
CMV319 FlowSOM Populations
US CD8
LAT CD8
LAT 4-1BB
LAT IL-10
Pop 0
Pop 1
Pop 2
Pop 3
Pop 4
Pop 5
Pop 6
Pop 7
B
CMV332 FlowSOM Populations

igure S6. FlowSOM Population tSNE overlays showing distribution. The output of the 8 clustered FlowSOM populations generated for donor CMV319 (A) and CMV332 (B) are visualized on the t-SNE plots for total unstimulated (US) and Latency associated proteins (LAT) stimulated CD8+ T cells and the 4-1BB expressing (LAT specific) and IL-10 secreting populations (LAT specific). The data presented here is summarized as the bar graphs in Figure 7 (A & D).

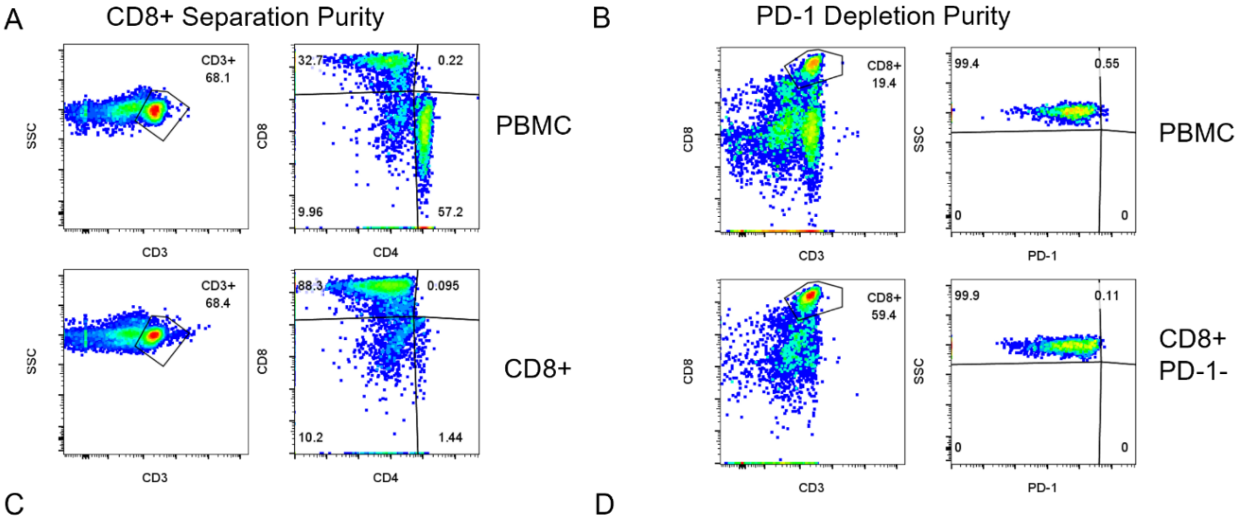

C

Donor	PBMC CD8%	PBMC CD4%	CD8+ CD8%	CD8+ CD8%
CMV319	32.7	57.2	88.3	1.44
CMV332	34.6	58.9	93.8	3.24

D

Donor	PBMC CD8%	PBMC CD8 PD-1%	CD8+ CD8%	CD8+ CD8 PD-1%
CMV319	19.4	0.55	59.4	0.11
CMV332	26.1	1.03	76.5	0.11

E

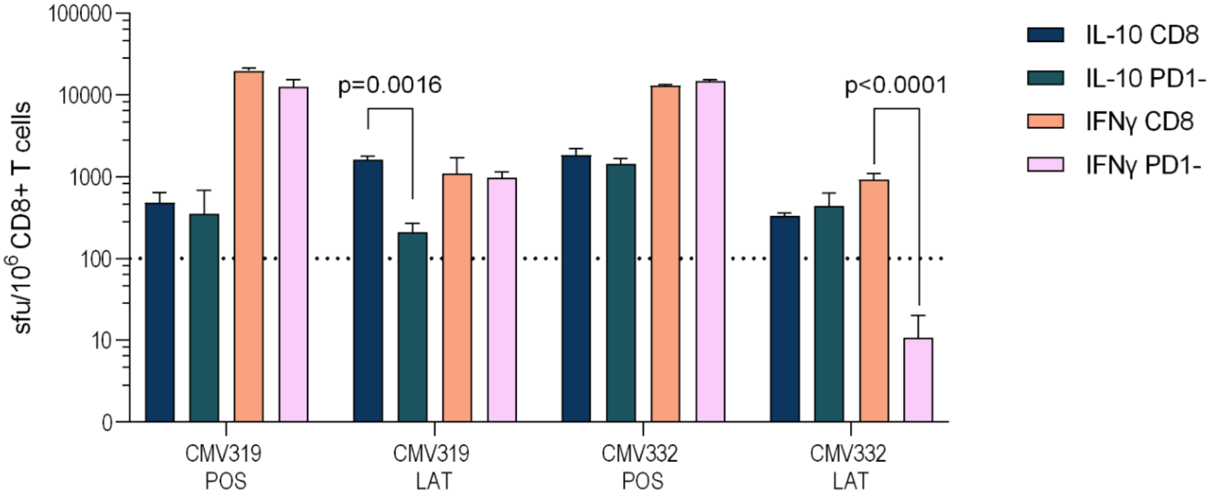

Figure S7. Depletion of PD-1 from resting CD8+ T cells results in varied production of IL-10 in response to HCMV peptide stimulation. Dual IFNγ and IL-10 fluorospot analysis of resting CD8+ T cells depleted of PD-1 expressing cells then stimulated by HCMV Latency associated proteins (LAT) from two HCMV sero-positive donors were performed. Representative flow cytometry dot plots showing the purity of the CD8

isolation (A) and the summarized data for donors CMV319 and CMV332 (C) are shown. The depletion of PD-1 expressing CD8+ T cells are shown in a representative dot plot (B) and the values for both donors are summarized (D). The background corrected IFNγ and IL-10 fluorospot response to the positive control (POS) and Latency associated proteins (LAT: UL138, US28, LUNA and vIL-10) mix stimulation for donors CMV319 and CMV332 are shown (E), with the positive response threshold of 100 sfu/million CD8+ T cells indicated (dotted line). Bars show the mean and standard deviation of the triplicate run for each cytokine and experimental condition. 1-way ANOVA with post hoc Šídák's multiple comparisons test were performed with significant differences ($p<0.05$) shown as values on the graph.

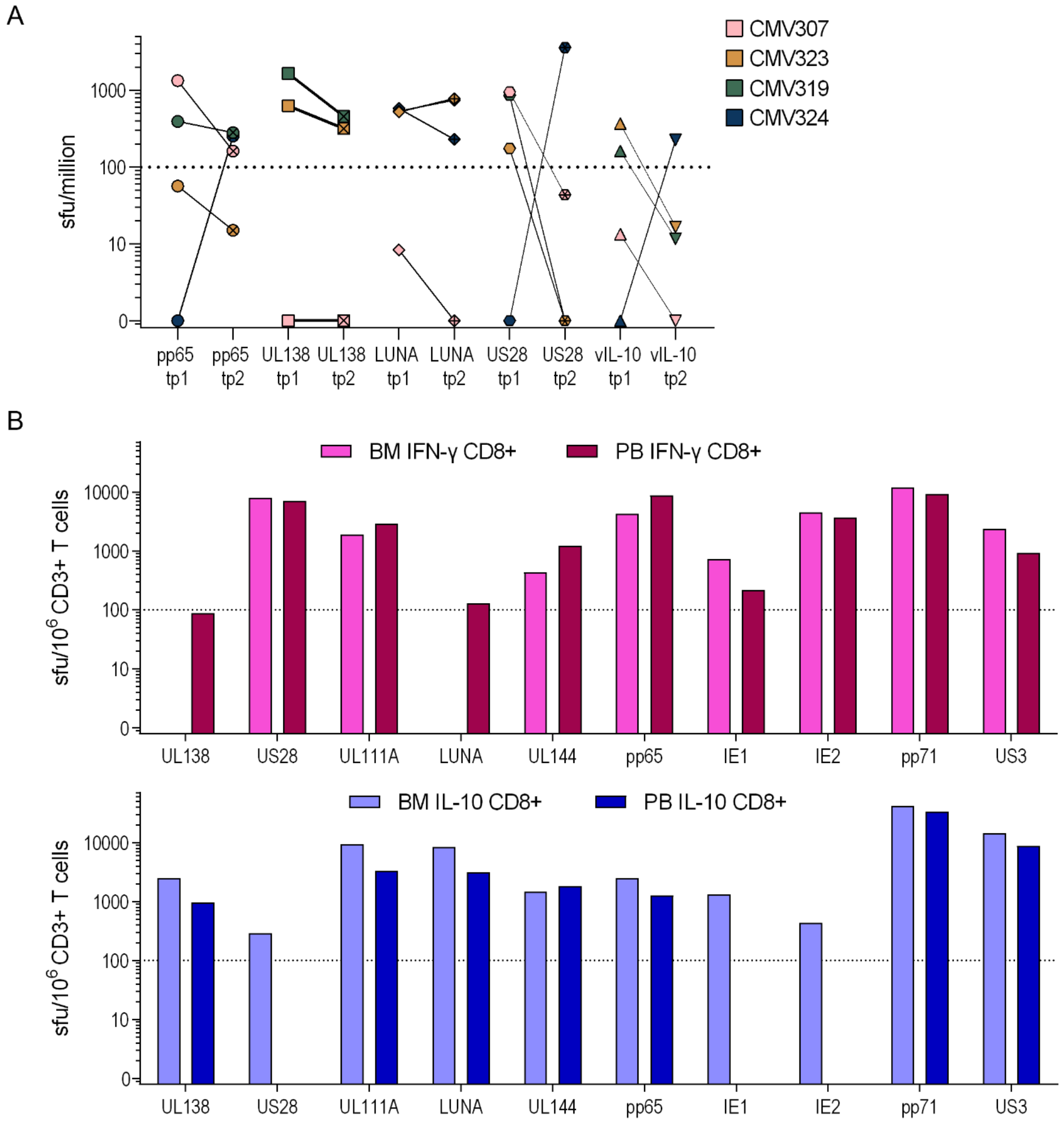

Figure S8. Fluctuation of IL-10 CD8+ T cell HCMV specific responses in peripheral blood and identification of CD8+ T cell IL-10 responses in the Bone Marrow. CD8+ T cells from 4 donors (CMV307 – pink symbols; CMV323 – yellow symbols; CMV319 – green symbols; CMV324 – blue symbols) were tested at two separate time points (tp1 & tp2) separated by 3 months (CMV319 and CMV323), by 4 months (CMV307) and 9 months (CMV324) (A). PBMC were depleted of CD4+ T cells and stimulated with overlapping peptide pools from pp65 (round symbol), UL138 (square symbol), LUNA (diamond symbol), US28 (hexagon symbol) and vIL-10 (triangle symbol) proteins for 48 hours on dual IFNγ and IL-10 Fluorospot plates. The results were converted to sfu/million T cells with background counts subtracted and the positive response threshold of 100 sfu/million indicated (dotted line). Between the two timepoints either the individual protein responses are present or absent (US28 and vIL-10) or there is fluctuation in the magnitude of the response detected (UL138 and LUNA). Paired peripheral blood (PB – dark bars) and bone marrow (BM – light bars) mononuclear cells depleted of CD4+ T cells were stimulated with 10 HCMV proteins (B); production of IFNγ (pink bars) and IL-10 (blue bars) was measured using an IFNγ and IL-10 fluorospot technique. Results were converted to sfu/million CD3+ T cells with background counts subtracted and the positive response threshold of 100 sfu/million CD3+ T cells is shown (dotted line) on each graph.
